# Photoactivatable Ruthenium Complexes Containing Minimal Straining Benzothiazolyl-1,2,3-triazole Chelators for Cancer Treatment

**DOI:** 10.1021/acs.inorgchem.3c04432

**Published:** 2024-02-22

**Authors:** Francisco J. Ballester, Alba Hernández-García, M. Dolores Santana, Delia Bautista, Pezhman Ashoo, Enrique Ortega-Forte, Giampaolo Barone, José Ruiz

**Affiliations:** † Departamento de Química Inorgánica, Universidad de Murcia and Biomedical Research Institute of Murcia (IMIB-Arrixaca), E-30100 Murcia, Spain; ‡ S.A.I., Universidad de Murcia, E-30100 Murcia, Spain; § Dipartimento di Scienze e Tecnologie Biologiche Chimiche e Farmaceutiche (SteBiCeF), Università degli Studi di Palermo, I-90128 Palermo, Italy

## Abstract

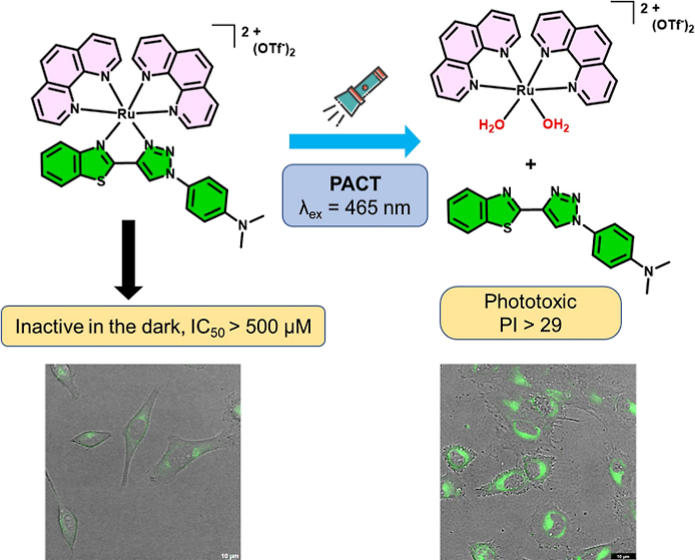

Ruthenium(II) complexes containing diimine ligands have contributed to the development of agents for photoactivated chemotherapy. Several approaches have been used to obtain photolabile Ru(II) complexes. The two most explored have been the use of monodentate ligands and the incorporation of steric effects between the bidentate ligands and the Ru(II). However, the introduction of electronic effects in the ligands has been less explored. Herein, we report a systematic experimental, theoretical, and photocytotoxicity study of a novel series of Ru(II) complexes **Ru1**–**Ru5** of general formula [Ru(phen)_2_(N^∧^N′)]^2+^, where N^∧^N′ are different minimal strained ligands based on the 1-aryl-4-benzothiazolyl-1,2,3-triazole (BTAT) scaffold, being CH_3_ (**Ru1**), F (**Ru2**), CF_3_ (**Ru3**), NO_2_ (**Ru4**), and N(CH_3_)_2_ (**Ru5**) substituents in the R4 of the phenyl ring. The complexes are stable in solution in the dark, but upon irradiation in water with blue light (λ_ex_ = 465 nm, 4 mW/cm^2^) photoejection of the ligand BTAT was observed by HPLC-MS spectrometry and UV–vis spectroscopy, with *t*_1/2_ ranging from 4.5 to 14.15 min depending of the electronic properties of the corresponding BTAT, being **Ru4** the less photolabile (the one containing the more electron withdrawing substituent, NO_2_). The properties of the ground state singlet and excited state triplet of **Ru1–Ru5** have been explored using density functional theory (DFT) and time-dependent DFT (TD-DFT) calculations. A mechanism for the photoejection of the BTAT ligand from the Ru complexes, in H_2_O, is proposed. Phototoxicity studies in A375 and HeLa human cancer cell lines showed that the new Ru BTAT complexes were strongly phototoxic. An enhancement of the emission intensity of HeLa cells treated with **Ru5** was observed in response to increasing doses of light due to the photoejection of the BTAT ligand. These studies suggest that BTAT could serve as a photocleavable protecting group for the cytotoxic bis-aqua ruthenium warhead [Ru(phen)_2_(OH_2_)_2_]^2+^.

## Abstract 2

The role of a series of new heteroleptic Ru(II) polypyridine complexes bearing BTAT chelators with different para substituents as photoassisted cytotoxicity agents under blue light against A2780 and HeLa human cancer cells has been investigated, as well as their electronic properties and photoejection of the BTAT ligand.

## Introduction

Cancer continues to be one of the main causes of death worldwide, only behind cardiovascular diseases.^1^ Different therapeutic approaches can be selected depending on their aggressiveness, stage, and accessibility, but in general terms, the standard strategy for malignant tumors usually involves resection of the tumor tissue, followed by localized radiotherapy and immunotherapy or chemotherapy. Systemic chemotherapy exhibits considerable limitations related to its typically low selectivity, which leads to numerous undesirable side effects, and drug resistances, relapses and metastatic tumors.^2,3^ Metal-based therapeutics, with their diverse coordination structures, and high tunability, possess unique properties, when compared to organic compounds.^3−10^ The use of the stimulus-responsive “prodrug approach” is very appealing to reduce the systemic toxicity,^11,12^ and in recent years, the photochemical and photophysical properties of precious metal complexes, such as strong spin–orbit coupling (SOC) effects, and tunable excited-state electronic configurations, have been exploited to make light-activated drugs for use as photodynamic therapy (PDT) and photoactivated chemotherapy (PACT) agents that allow to minimize effects on normal tissue through the use of light directed to the tumor, achieving a high temporal and spatial control.^13−23^ PDT requires the combination of three fundamental components, namely, a nontoxic photosensitizer (PS), ground state molecular oxygen (^3^O_2_), and light, to generate highly toxic singlet oxygen (^1^O_2_) in a photocatalytic manner and subsequently to induce cancer eradication with low systemic toxicity.^24^ Thus, it is important to highlight that the polypyridyl Ru(II) complex TLD-1433, reported by McFarland and co-workers,^25^ is being currently studied as a photosensitizer for PDT in Phase II clinical trials for the treatment of nonmuscle invasive bladder cancer (NMIBC). However, despite recent promising research advances,^18,26−30^ due to the hypoxic nature of many native tumors, PDT is frequently limited in its therapeutic effect.^31−33^ Additionally, oxygen consumption during PDT may exacerbate the tumor’s hypoxic condition, which stimulates tumor proliferation, metastasis, and invasion, resulting in poor treatment outcomes.^34^ Compared with traditional PDT, PACT offers an oxygen-independent mechanism, requiring only a photosensitive complex and light to operate, being therefore more suitable for hypoxic tumors.^35−37^ In addition, PACT can be a useful technique to deliver already known chemotherapeutic or bioactive agents, notably in the case of enzyme inhibitors,^38^ where the effect of the desired molecule is directed only to its target after light irradiation.^39,40^

Octahedral bis- and tris-heteroleptic ruthenium(II) scaffolds represent a highly promising family of uncaging molecules via a photosubstitution reaction, due in part to their ease of modification and tunability as well as their biological and photophysical profiles.^29,39,41,42^

For most ruthenium-based PACT agents, once the complex is excited to its triplet metal-to-ligand charge transfer (^3^MLCT state), it rapidly populates the metal-centered triplet (^3^MC) state, which removes the ruthenium photocage. The octahedral Ru(II) systems proposed for PACT are often based on the incorporation of distortion into the structure of the coordination complex through the use of, for example, hindering polypyridyl ligands, lowering the energy of dissociative excited states, and increasing the yield of the photosubstitution reaction.^37,42−44^ Alternatively, the photorelease of monodentate ligands can be easier, as they are not subject to the chelate effect.^45−47^

**Figure 1:**
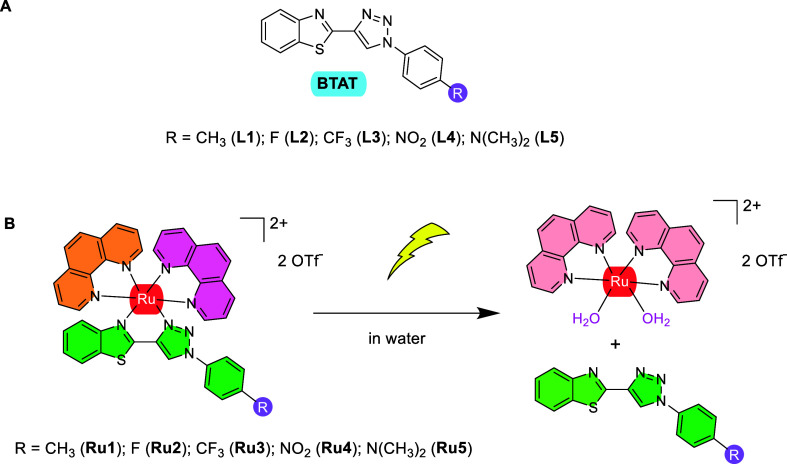
BTAT ligands (A). New synthesized ruthenium complexes and possible photosubstitution reaction when complex is irradiated with visible light in water (B).

On the other hand, benzothiazoles represent privileged scaffolds in medicinal chemistry with many applications as anticancer agents.^48^ Thus, the 5-fluorobenzothiazole prodrug (Phortress) is a suitable candidate for Phase I clinical trials.^49^ We have recently reported a series of benzothiazolyl-1,2,3-triazole molecules (**L1–L5**, Figure 1A), possessing a push–pull architecture, and exhibiting moderate to high selective antiproliferative activity in A2780 and HeLa cancer cells, together with interesting optical properties based on charge-transfer emission depending on the substituent in the 1,2,3-triazole moiety.^50^ Based on this background, herein, we developed a series of novel photoactive Ru(II) octahedral complexes containing benzothiazolyl-1,2,3-triazole (BTAT) chelators lacking steric hindrance (**Ru1–Ru5**, Figure 1B) in order to explore their potential action as phototherapeutic anticancer agents, together with their photochemistry and theoretical calculations.

## Results and Discussion

### Synthesis and Characterization of Ru(II) Complexes (**Ru1–Ru5**)

The benzothiazolyl-1,2,3-triazole ligands **L1–L5** (Figure 1A) were prepared by condensation reactions between the respective 1,2,3-triazole-4-carbaldehydes and *ortho*-aminothiophenol, as recently reported by us (Scheme S1).^50^ Important to note, these BTAT derivatives allowed intramolecular charge transfer tuning, apart from exhibiting solvatofluorochromism and selective antiproliferative properties, whereas their coordination chemistry is still unexplored.

**Scheme 1:**
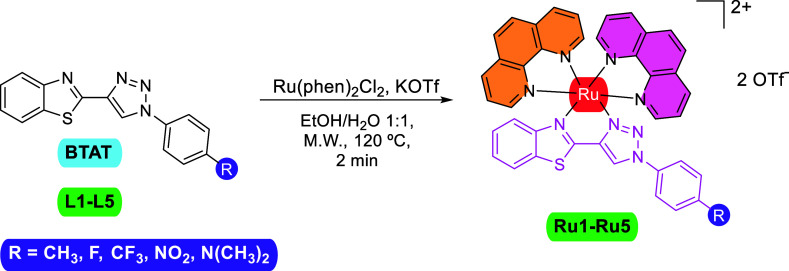
Synthesis of Complexes **Ru1–Ru5**

The synthesis of the new orange air- and moisture-stable complexes **Ru1–Ru5** was carried out (Scheme 1) by the reaction of Ru(phen)_2_Cl_2_ with the corresponding BTAT ligand in an ethanol–water mixture (1:1) and potassium triflate under microwave in 2 min. **Ru1–Ru5** complexes were characterized using multinuclear ^1^H and ^13^C{^1^H} NMR spectroscopy (Figures S1–S32 in the Supporting Information). Final evidence of the correct formation of the compounds has been obtained from the high-resolution ESI^+^ mass spectra (Figures S33–S37).

**Chart 1:**
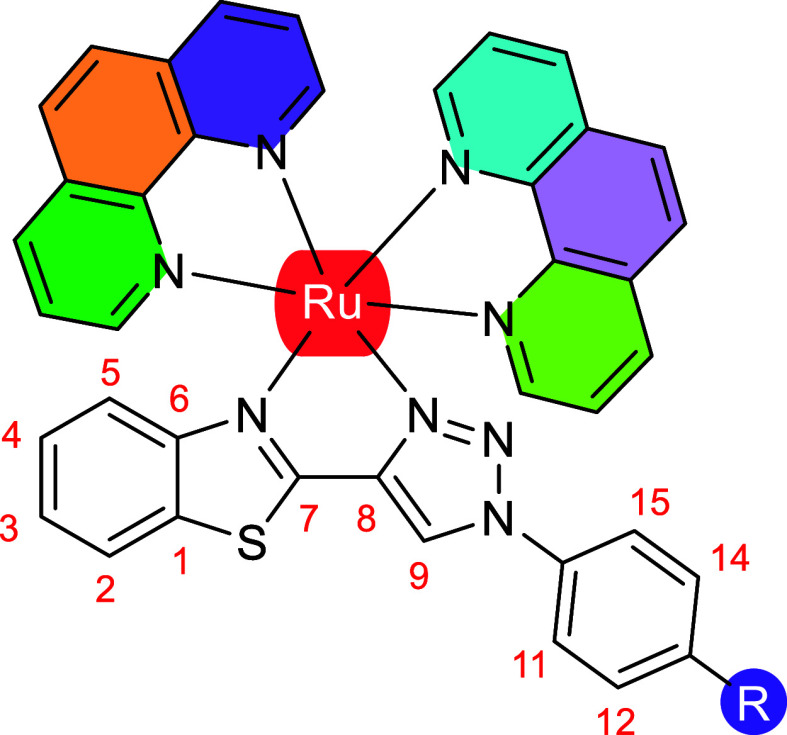
Hydrogen and Carbon Labels Used for BTAT Ligands in ^1^H NMR Assignments of Complexes **Ru1–Ru5**

**Figure 2:**
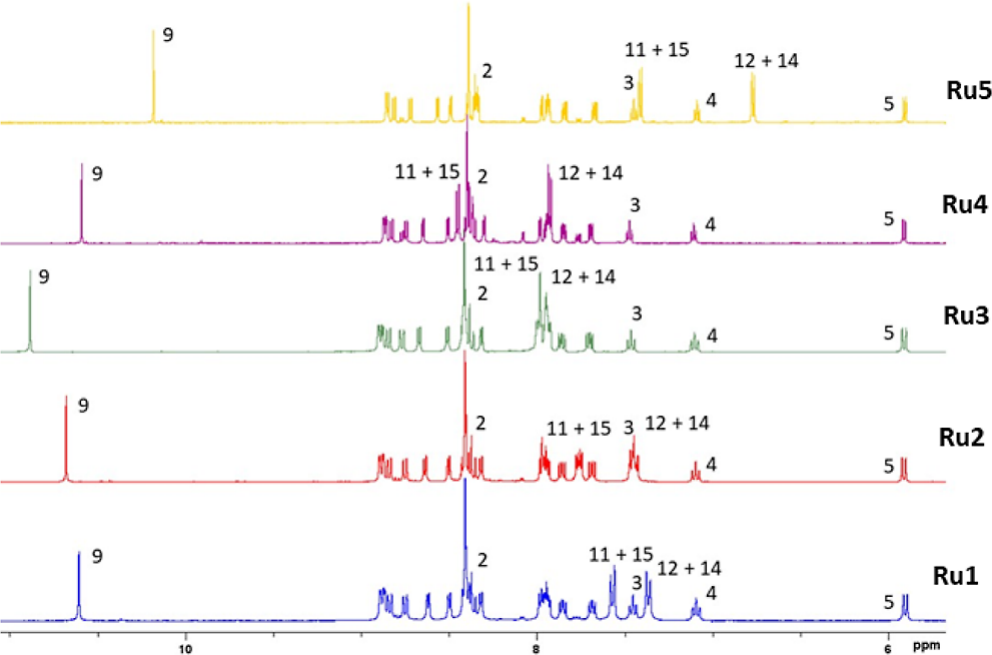
^1^H NMR spectra of complexes **Ru1–Ru5** in DMSO-*d*_6_ at 293 K, aromatic region.

Hydrogen and carbon labels used in ^1^H NMR assignments of complexes **Ru1–Ru5** are shown in Chart 1. In the ^1^H NMR spectra, the signals of the aromatic protons appear between 5.5 and 11 ppm, being the proton H9 of the 1,2,3-triazole ring (Chart 1) which appears at more downfield as a singlet resonance. In the aliphatic region of the ^1^H NMR spectra, only signals from complexes **2** and **5** are observed, due to the CH_3_ and N(CH_3_)_2_ substituents on the phenyl ring of the 1-(aryl)-4-(benzothiazolyl)-1,2,3-triazole, respectively. The stacked ^1^H NMR spectra of all complexes for comparison is shown in Figure 2.

^1^H NMR signals of the benzothiazole fragment (H2–H5) appear as two doublets (H2 and H5) and two pseudotriplets (H3 and H4). The resonances of the protons of the phenyl ring H11, H12, H14, and H15 appear as doublets at different chemical shift values since they are influenced, like the H9 proton, by the electron-donating or electron-withdrawing capacity of the substituent on the ligand 1-aryl-4-benzothiazolyl-1,2,3-triazole. With these characteristic signals as starting point, the resonances of the ^1^H and ^13^C NMR were assigned via the observed ^1^H–^1^H COSY, ^1^H–^1^H NOESY, ^13^C–^1^H HSQC, and ^13^C–^1^H HMBC correlations (Figures S1–S32). The resonances of the benzothiazole fragment of the H2 and H5 protons, the most shielded and the most unshielded, respectively have been assigned from the ^13^C–^1^H HMBC spectra. In these spectra, the correlations of C1 with H2, C6 with H5, and C8 with H9 were observed. From these assignments, the resonances of all the protons and carbons of the BTAT ligands can be assigned and are listed in Table S9. These assignments are also supported by the ^1^H–^1^H NOESY spectra in which correlations between the H5 proton and phen rings protons are observed.

### X-ray Crystallography

**Figure 3:**
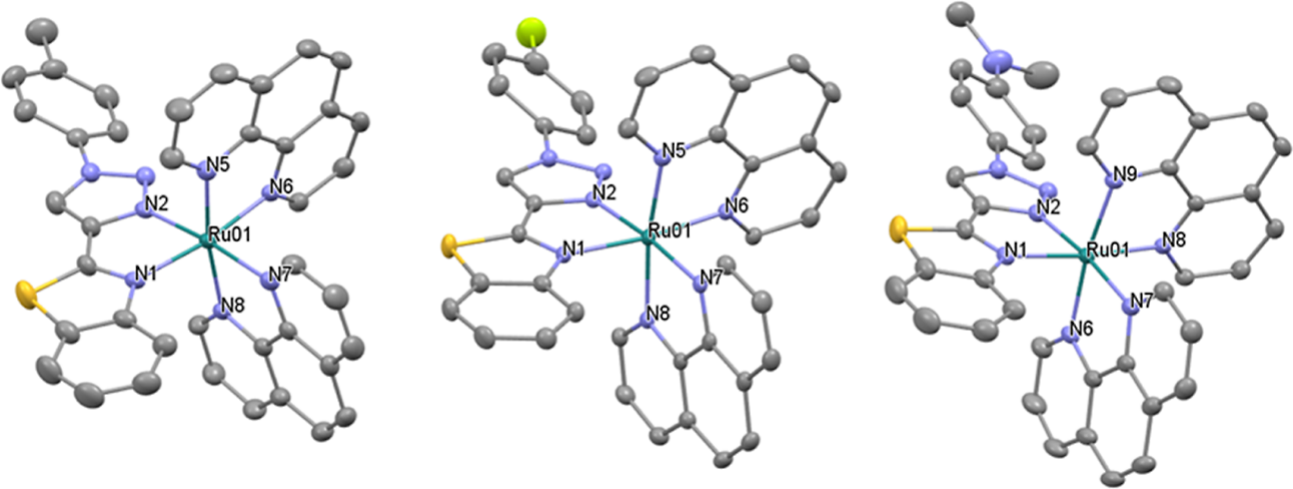
ORTEP plots of the cations of complexes **Ru1** (left), **Ru2** (middle), and **Ru5** (right). For clarity, counterions and hydrogen atoms have been omitted. Ellipsoids have been represented at 50% probability. CCDC reference numbers are 2284059 for **Ru1**, 2284060 for **Ru5**, and 2284061 for **Ru2**.

The coordination geometries of cations in complexes **Ru1**, **Ru2**, and **Ru5** were confirmed by single-crystal X-ray crystallography (Figure 3 and Table S1). The complexes crystallize in the triclinic space group *P*1̅. The ORTEP plots of the structures of the cations are shown in Figure 3 and Table 1 contains selected bond lengths and angles. There are two interstitial ethanol molecules in the asymmetric unit of **Ru2** and one acetonitrile molecule in the asymmetric unit of **Ru5**. The cations exhibit distorted octahedral geometries with ruthenium–nitrogen (phen) bond lengths similar to the values reported for a ruthenium(II) tris–diimine complex with values between 2.044 and 2.067 Å for the two phen ligands.^51a^ The bond lengths Ru–N1 (benzothiazole moiety) are longer (approximately 2.11 Å), while the Ru–N2 (triazole moiety) are the shortest (approximately 2.03 Å),^51b^ maybe due to the fact that the smaller ring size for a triazole donor and the absence of a C–H proton adjacent to the coordinating N atom, which is present for other N-heterocyclic ligands, and making the triazole donor less sterically demanding. The N–M–N bite angles range from 77.4° for BTAT to 80.2° for phen, being the values found for the BTAT ligands similar in the three complexes. The dihedral angles N1–C7–C8–N2 are −1.47, −0.81, and −0.22° for complexes **Ru1**, **Ru2**, and **Ru5**, respectively, showing the quasi-planar coordination of the BTAT ligands. Apart from the cation–anion triflate Coulomb interactions, the packing in the structures of **Ru1**, **Ru2**, and **Ru5** are organized by C–H···N, C–H···O, and O–H···O intra- and intermolecular interactions (Tables S2 and S3 and Figures S38–S39). Intermolecular π–π interactions involving the phen rings are also observed (Figures S40–S42). The usual π-interaction is an offset or slipped stacking and the ring normal and the vector between the ring centroids form an angle of about 20° up to centroid–centroid distances of 3.8 Å.^52^ As all the π–π interactions in our compounds have shorter centroid distances (3.5300(12) to 3.7770(17) Å; Table S4) and the angle between the ring normal and the vector Cg–Cg is in the range of 19.2 to 28.9° (Table S4), the π–π interactions in these compounds belong to strong π–π interactions. In these complexes, the π–π interactions form the chains along the *c* axis.

**Table 1:** Selected Bond Lengths (Å) and Angles (deg) for Complexes **Ru1**, **Ru2**, and **Ru5**

	**Ru1**	**Ru2**	**Ru5**
Ru(01)–N(1)	2.117(2)	2.1167(18)	2.1117(14)
Ru(01)–N(2)	2.027(2)	2.0359(18)	2.0374(14)
Ru(01)–N(5)^a^	2.063(2)	2.0678(19)	2.0622(14)
Ru(01)–N(6)^b^	2.044(2)	2.0501(18)	2.0677(14)
Ru(01)–N(7)^c^	2.059(2)	2.0651(18)	2.0451(13)
Ru(01)–N(8)^d^	2.065(2)	2.0607(19)	2.0666(13)
N(1)–Ru(01)–N(2)	77.53(9)	77.91(7)	77.36(5)
N(5)^a^–Ru(01)–N(6)^b^	79.68(9)	79.95(7)	79.90(5)
N(7)^c^–Ru(01)–N(8)^d^	80.14(9)	80.17(7)	79.88(5)

a In complex **Ru5**: N(6).

b In complex **Ru5**: N(7).

c In complex **Ru5**: N(8).

d In complex **Ru5**: N(9).

### Photophysical Properties

**Figure 4:**
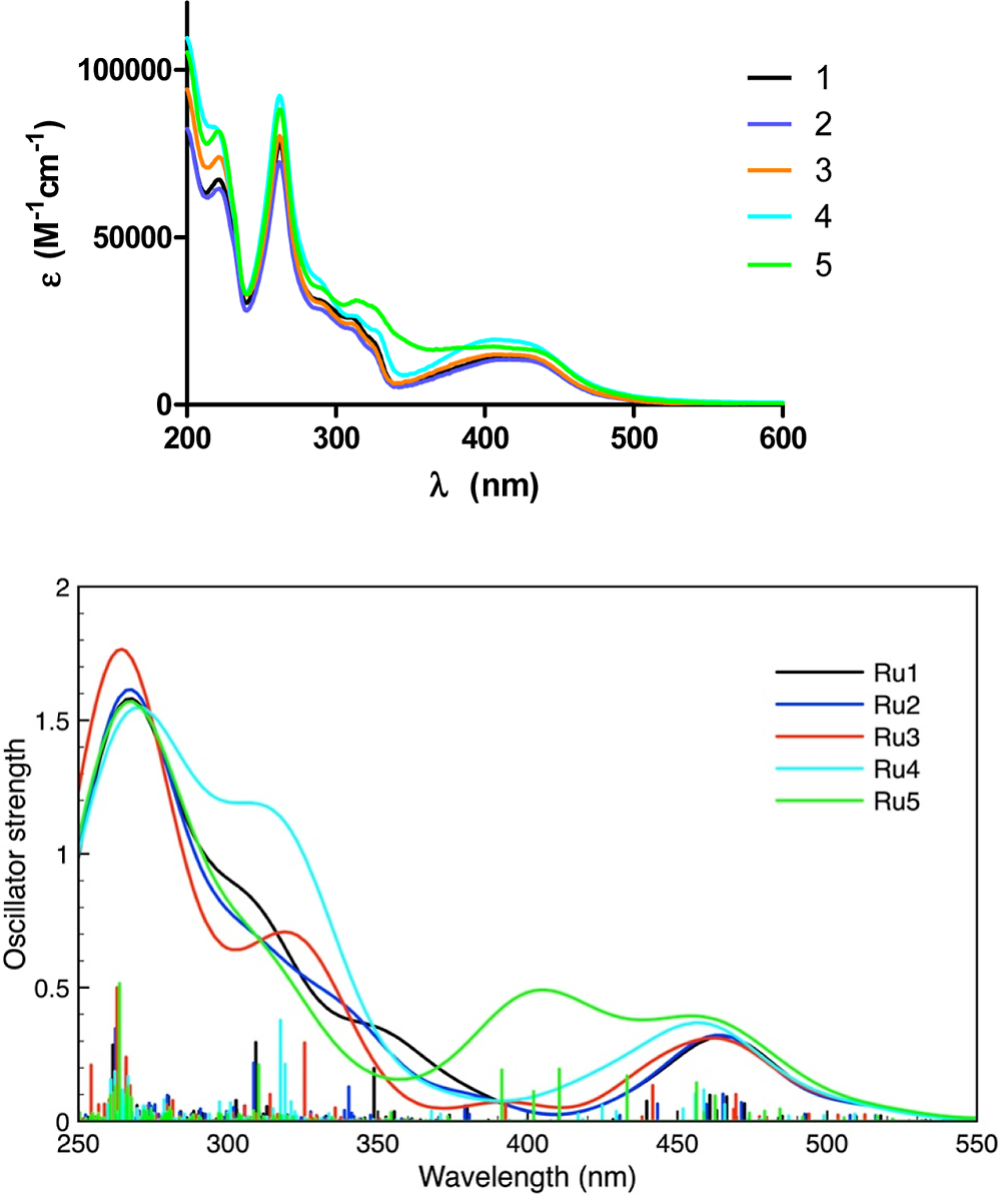
Experimental (top) and calculated (bottom) UV–vis absorption spectra of complexes **Ru1–Ru5** in H_2_O.

**Figure 5:**
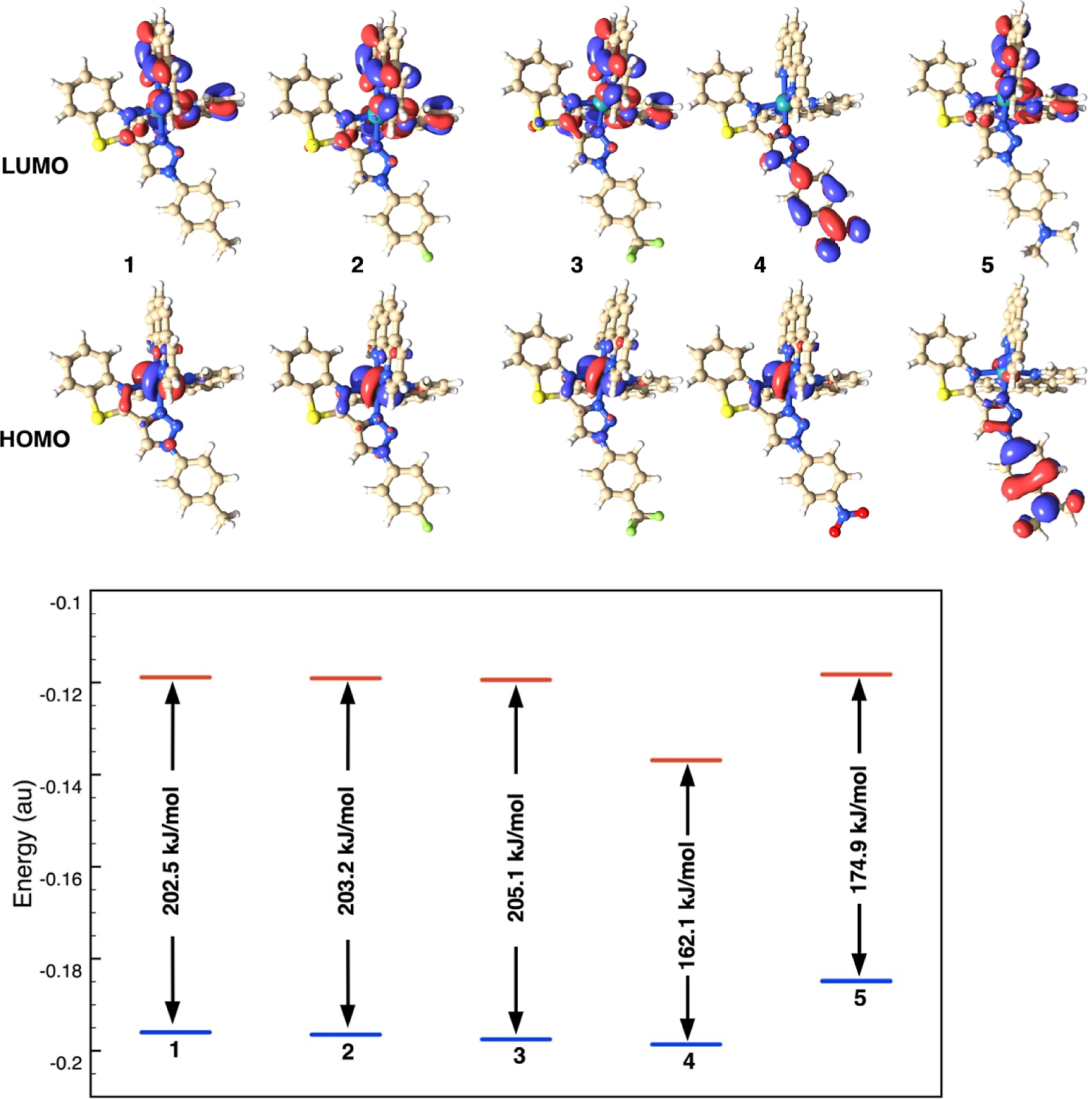
HOMO–LUMO orbitals of the Ru complexes **Ru1**–**Ru5** in H_2_O, singlet ground state, S_0_, obtained by DFT calculations (top). Energies (in au) and energy gaps (in kJ/mol) of the HOMO and LUMO of compounds **Ru1–Ru5**, obtained by DFT calculations in H_2_O solution (bottom).

The UV–visible absorption spectra of complexes **Ru1**–**Ru5** have been recorded in ACN and water solutions 10^–5^ M (Table 2 and Figures 4 and S43 and S54). All complexes display sharp and intense bands in the region below 350 nm corresponding to singlet intraligand ^1^ππ* transitions that are allocated to the polypyridyl and BTAT ligands. On the other hand, the broad bands of lower intensity between 400 and 500 nm could be assigned to singlet metal-to-ligand charge transfer transitions (^1^MLCT), from dπ orbitals of Ru to the π* orbitals of the ligand(s). Our complexes contain two phen ligands and the BTAT ligand contains a delocalized π system that in some cases could have an intraligand charge transfer (ILCT) character due to the more polarizing groups. Therefore, as expected, the absorption spectra showed characteristics of the BTAT ligands. The calculated UV–vis spectra (Figure 4 bottom, in water, and Figure S54, in ACN) were found to be in accordance with the experimental data (Figure 4 top). Observing the absorption spectra along the series of complexes **Ru1–Ru5** allows us to conclude that the contributions of the BTAT ligands to the LUMO in complexes **Ru1–Ru3** is very small and therefore these orbitals are mainly delocalized on the other N^∧^N ligands (phen). The differences are greater in the spectra of complexes **Ru4** and **Ru5** that are blue-shifted, which could indicate a greater contribution of the BTAT ligands in the LUMO or in HOMO, respectively (see below, Figure 5).

**Table 2:** UV–Visible Absorption Data for Complexes **Ru1–Ru5**

complex		λ_abs_/nm (ε/dm^3^ mol^–^^1^ cm^–^^1^)
**Ru1**	ACN	224 (65,150), 263 (76,690), 293 sh (29,850), 313 sh (25,640), 327 sh (18,700), 416 (14,200)
	H_2_O	221 (67,110), 262 (77,840), 294 sh (29,950), 312 sh (25,710), 322 sh (20,200), 417 (14,280)
**Ru2**	ACN	224 (71,110), 263 (80,960), 293 sh (30,070), 312 sh (25,030), 327 sh (16,880), 415 (14,910)
	H_2_O	221 (64,380), 262 (72,390), 290 sh (28,400), 310 sh (22,660), 324 sh (16,240), 423 (13,380)
**Ru3**	ACN	224 (74,470), 263 (81,440), 293 sh (29,230), 312 sh (24,580), 326 sh (17,780), 410 (15,100)
	H_2_O	221 (73,820), 262 (80,270), 290 sh (30,330), 314 sh (23,340), 325 sh (17,380), 417 (14,900)
**Ru4**	ACN	222 (70,220), 263 (83,260), 290 sh (30,920), 314 sh (22,530), 329 sh (18,700), 406 (16,870)
	H_2_O	219 (82,830) sh, 262 (92,260), 288 sh (37,510), 313 sh (26,480), 326 sh (22,210), 407 (19,420)
**Ru5**	ACN	221 (7500), 264 (80,630), 316 sh (27,370), 325 sh (27,000), 345 sh (21,660), 433 (15,360)
	H_2_O	221 (81,490), 262 (88,120), 291 sh (34,630), 314 sh (31,160), 406 (17,390)

The complexes **Ru1–Ru3** present dual emission at room temperature when the excitation was made by light with the appropriate wavelength see Table 3 and Figure S44. Fluorescence in the ultraviolet region corresponding to ^1^IL states and weak yellow phosphorescence from ^3^MLCT states. This behavior has been previously observed in Ru(II) complexes.^53−57^

**Table 3:** Luminescence Data, ^1^O_2_ Generation Quantum Yields (Φ_Δ_), and Half-Life (*t*_1/2_) for Photoejection^f^ for Complexes **Ru1–Ru5**

complex	λ_em_, nm (λ_exc_)^a^	τ_em_, ns (%)^b^	Φ_P_ (λ_em_/nm)^b^^,^^c^	Φ_Δ_ (air)^d^	*t*_1/2_ (min)^e^
**Ru1**	362 (305)	8.4 (56); 275 (44)	<0.01 (532)	0.02	4.85
	532 (390)				
**Ru2**	362 (305)	1.8 (3); 419 (97)	<0.01 (579)	0.02	4.55
	579 (400)				
**Ru3**	370 (320)	7.8 (83); 202 (17)	0.01 (514)	0.03	5.54
	514 (320)				
**Ru4**	517 (320)	7.9 (88); 171 (12)	<0.01 (517)	0.04	14.15
**Ru5**	516 (320)	7.1 (80); 95 (20)	0.09 (516)	0.01	6.78

a In ACN solutions, 294 K.

b In ACN solutions, 294 K, Ar.

c Absolute emission quantum yield.

d Reference [Ru(bpy)_3_]^2+^: ACN Φ_Δ_ = 0.56.^58^

e In water.

f Measured using a 3 mW cm^–2^ 465 nm LED.

The ^1^IL fluorescent emission observed at 362 nm in ACN for complexes **Ru1** and **Ru2** was more intense than that observed for complex **Ru3**. All the complexes present weak yellow phosphorescence from ^3^MLCT states at room temperature in ACN solutions, see Table 3. The emission from ^3^MLCT varies little from one compound to another, which could be indicate of the π* acceptor orbital of ^3^MLCT is similar in each complex. Although the emission intensity around 500 nm is not very intense, the decay profile of the lifetime of the excited state was found to be biexponential in nature. The biexponential decay shows a short (2–8 ns) and a long (95–420 ns) component. This biexponential decay indicates that multiple triplet excited states are involved in the emission profile.

### DFT Calculations

The properties of the ground state singlet and excited state triplet of complexes **Ru1–Ru5** have been explored using DFT and TD-DFT calculations. Optimized geometries were obtained in ACN and water. The structures of the singlet ground and triplet excited states, in water, are shown in Figures S52–S53. The structures obtained in ACN are essentially the same as those obtained in water. Interestingly, for all **Ru1–Ru5** complexes, the N1–Ru distance, between the Ru atom and the BTAT nitrogen, is consistently larger in the T_1_ state, about 0.49 Å, compared to that in the S_0_ state (Table S5). This indicates that the excitation to the triplet spin state induces a weakening of such a bond. The electronic structures of the ground state of the complexes were characterized according to their frontier molecular orbitals HOMO and LUMO, Figure 5 top. The nature of the HOMO changes with the substituents of the BTAT ligand, as can be seen in Figure 5. Thus, the HOMO of complexes **Ru1–Ru4** was predominantly ruthenium d-orbital character but with some additional BTAT π-contribution and in complex **Ru5** was instead located on the triazole rings and its phenyl–N(CH_3_)_2_. The LUMO was dominated by π* contribution from the phen ligands in complexes **Ru1–Ru3**. However, in complex **Ru4** it comprises the triazole ring and its 4-NO_2_-phenyl substituent.

The LUMO was progressively stabilized from **Ru5**, **Ru1**, **Ru2**, **Ru3**, to **Ru4** as the electron-withdrawing character of the substituent of BTAT ligands increases (Figure 5 bottom). The HOMO was also stabilized in similar extent leading the shorter HOMO–LUMO gap for **Ru4** (164.5 kJ/mol) following by **Ru5** (173.6 kJ/mol) and compared to **Ru1–Ru3** (202–205 kJ/mol) (Figure 5 bottom). Very similar orbital shapes and energy values were obtained also in ACN.

In all complexes, the T_1_ state mainly originates from HOMO → LUMO (70%) transition (Table S6) and characterized as ^3^MLCT. The other high energy emission band are derived from triplet states which are predominantly ligand centered ^3^LLCT/^3^ILCT.

Finally, it is interesting to note that the S_0_–T_1_ transition is accompanied by a change in the dipole moment (Table S8), which is greater for **Ru5** and **Ru1**, in particular in water and **Ru4** exhibited the lowest change in the dipolar moment in the S_0_–T_1_ transition.

### Photochemistry Studies

The capacity of complexes **Ru1–Ru5** to undergo photosubstitution reactions was explored by UV–vis. In the dark, UV–vis measurements revealed a remarkable stability in water at 310 K of **Ru1–Ru5** over a period of 120 h, as shown in Figure S45 no changes in their UV–vis spectra were observed.

**Figure 6:**
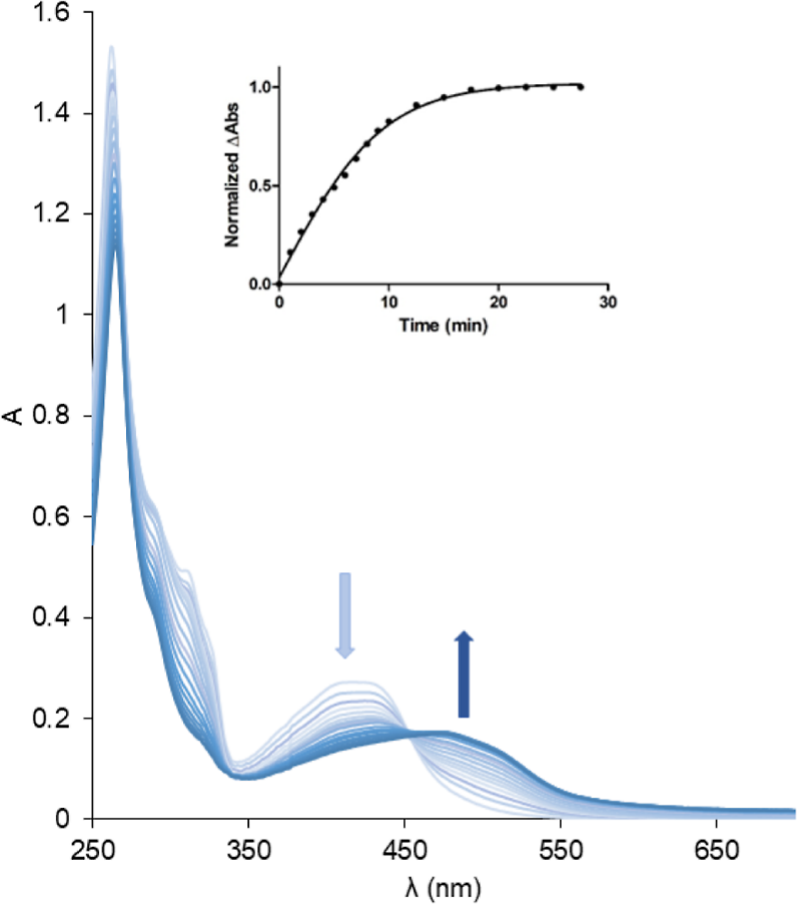
Changes in absorption spectra of **Ru1** in H_2_O (10^–5^ M) as observed upon irradiation with blue light (λ_ex_ = 465 nm, 4 mW/cm^2^).

Conversely, light irradiation (λ = 465 nm, 4 mW/cm^2^) of water solutions of the new complexes provokes with the time noticeable changes in their absorption spectra, as can be seen in Figure 6 for **Ru1** and in Figure S46 for complexes **Ru2–Ru5**. Upon light irradiation, the metal to ligand charge transfer (MLCT) band centered between 410 and 425 nm was bathochromically shifted to a broad MLCT band centered at 478 nm, with time a clear isosbestic point around 450 nm was observed along with decreasing absorption intensity within 250–350 nm.

Parallel HPLC experiments for the Ru complexes (vide infra) evidenced the existence of a photoejection process of the corresponding BTAT ligand (Figures S47–S51 for **Ru1–Ru5**). The evaluation of these changes in the absorption spectra demonstrated variation in *t*_1/2_ values through all complexes (Figure 6 and Table 3).

Under these conditions complexes **Ru1–Ru3** became more labile, *t*_1/2_ between 4.5 and 5.5 min, instead complex **Ru4** containing the BTAT ligand with the more electron-withdrawing substituent (−NO_2_) was the less active, *t*_1/2_ = 14.15 min. Complex **Ru5** with BTAT ligand with the substituent more electron donating [−N(CH_3_)_2_] shows *t*_1/2_ around 6.8 min. Except for complex **Ru4**, these complexes exhibit similar efficient photosubstitution than other complexes with bidentate ligands and steric hindrance which have *t*_1/2_ < 5 min. In these photolabile compounds the ^3^MLCT excited states generated photochemically are quenched by low lying metal-centered (^3^MC) triplet excited states that lead to nonradiative decay and photosubstitution.^59,60^

**Figure 7:**
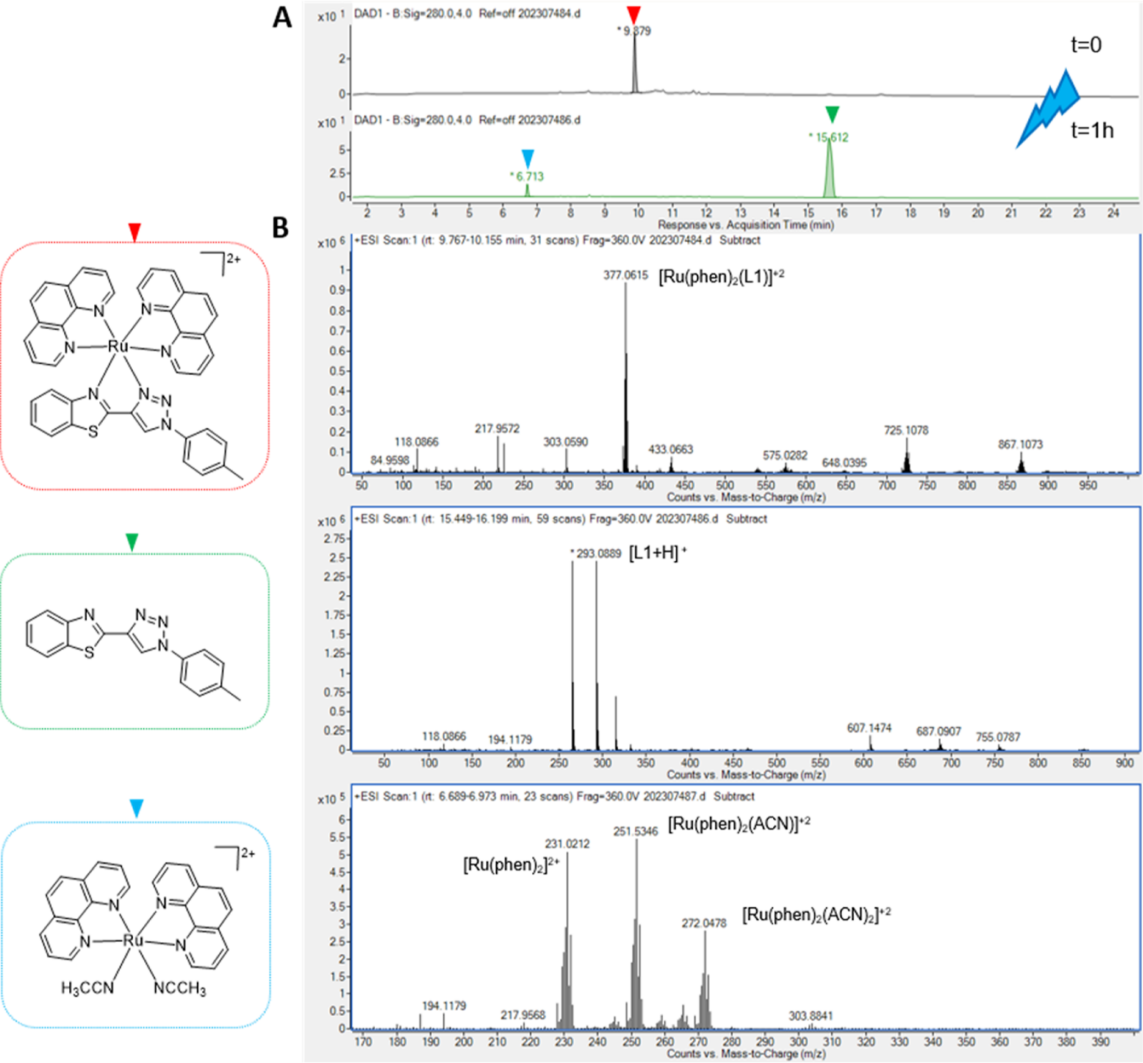
Determination of photoejection products of **Ru1** by HPLC-MS. (A) HPLC chromatogram (DAD = 280 nm) of **Ru1** at *t* = 0 (top) and after being irradiated for 1 h with blue light, λ_ex_ = 465 nm, 4 mW/cm^2^. (B) Mass spectra of the peaks of interest extracted from the chromatograms.

As stated above, the release of the BTAT ligand from the corresponding prodrug **Ru1–Ru5** could be confirmed by HPLC using ACN solutions of the complexes at time zero and after 1 h of irradiation with blue light (λ_ex_ = 465 nm, 4 mW/cm^2^) (Figures S47–S51). As observed in Figure 7A, the chromatograms show the disappearance of complex **Ru1** peak (*t*_R_ = 9.9 min), while a new signal appears at a higher retention time (*t*_R_ = 15.6 min) corresponding to the BTAT ligand, together with the peak at a lower retention time (*t*_R_ = 6.7 min), assigned to the Ru photoproduct [Ru(phen)_2_(ACN)_2_]^2+^ (calcd *m*/*z* = 272.05, Figure 7B), a labile complex that could potentially bind to DNA or other biomolecules within the cell.^61^

**Figure 8:**
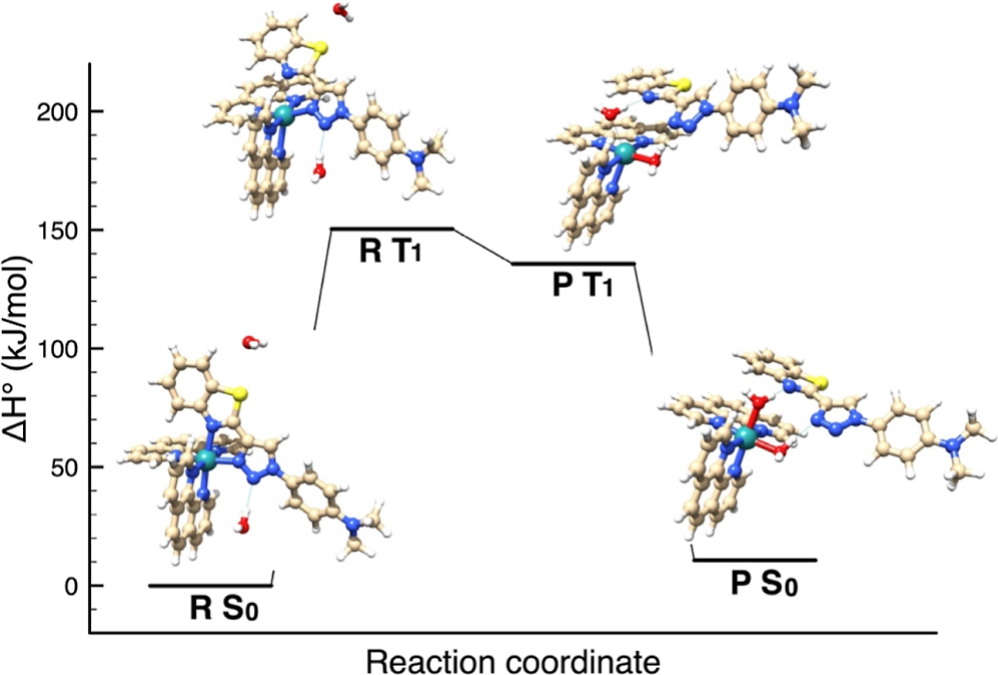
Proposed mechanism of ligand photoejection for complex **Ru5**, in H_2_O, obtained by DFT calculations.

The remarkable N1–Ru bond weakening in the triplet state (Table S5) suggests a possible mechanism involved in the photoejection of the BTAT ligand. An example of such a mechanism, considering compound **Ru5**, is shown in Figure 8. The proposed mechanism of ligand photoejection for complex **Ru5**, in H_2_O, was obtained by DFT calculations. The ground state reactant, S_0_, in the presence of two water molecules, is excited into the triplet state T_1_; in the excited state, the two water molecules substitute the chelating ligand and finally the substitution product decays from T_1_ into the ground state S_0_. Analogous result is obtained for compound **Ru4** (Figure S55), showing that the slower reactivity observed of compound **Ru4** is attributable to the larger *t*_1/2_ photoejection half time (Table 3).

For ruthenium polypyridyl compounds photosubstitution reactions compete with phosphorescence and ^1^O_2_ generation.^62^ The capacity of **Ru1–Ru5** to generate ^1^O_2_ upon irradiation at 465 nm (4 mW/cm^2^) in aerated CH_3_CN was examined by measuring the absorption of 1,3-diphenylisobenzofuran (DPBF) at 413 nm in acetonitrile solution. The complexes showed comparatively low but nonzero Φ_Δ_ values (Table 3), suggesting that a PDT type II mechanism can hardly explain the phototoxicity observed in normoxic conditions.^63^ Important to note, the related bis-aqua ruthenium complex [Ru(Ph_2_phen)_2_(H_2_O)_2_]^2+^ has been probed able to bind to proteins or nucleic acids and subsequently generate ROS.^63^

### Photobiological Studies

#### Cell Uptake

**Figure 9:**
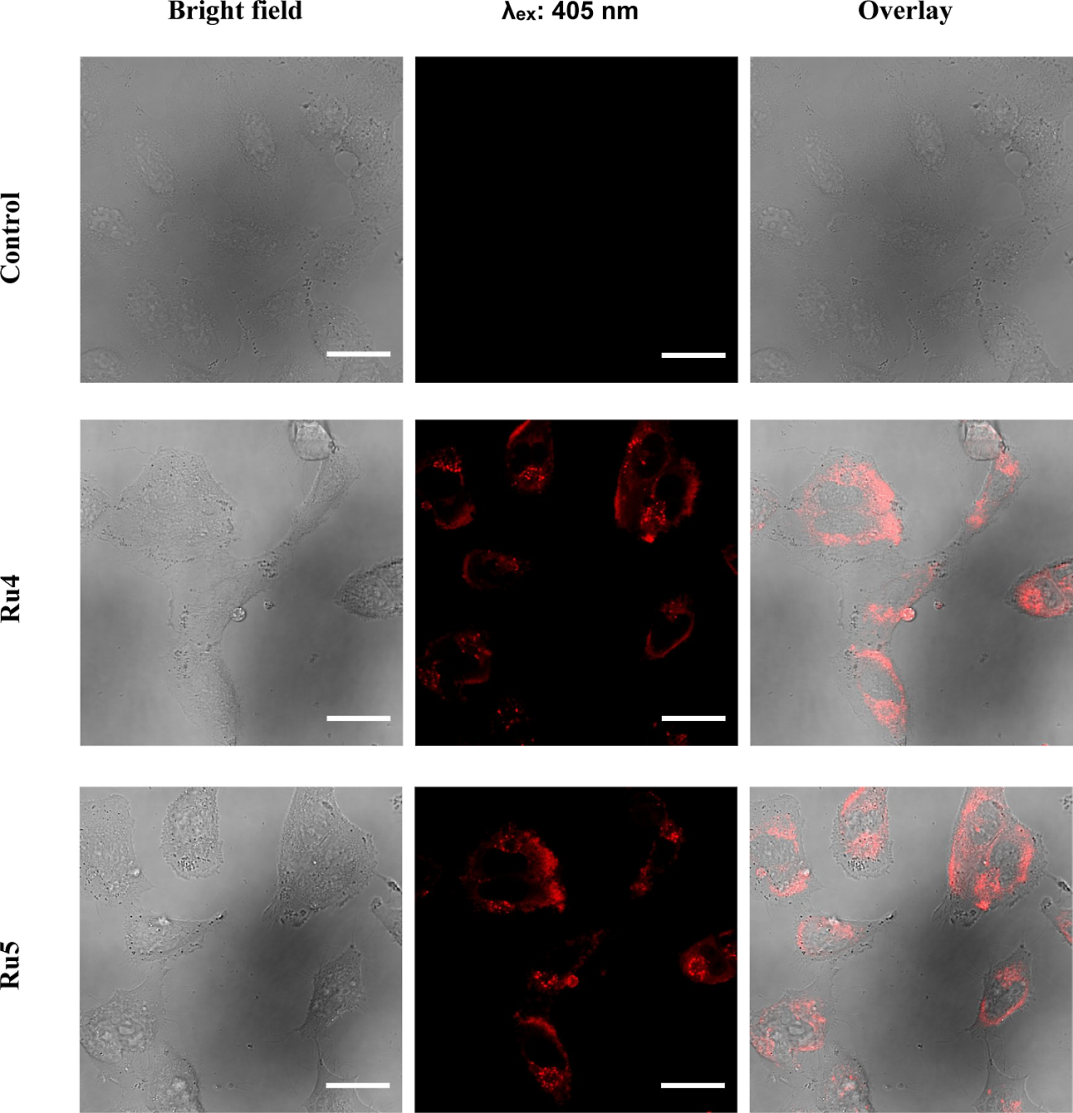
Confocal microscopy images of HeLa cells incubated with Ru(II) complexes **Ru4** and **Ru5** [15 μM] for 2 h. Scale bar 30 μm.

First, the cellular uptake of two representative Ru(II) complexes were investigated in HeLa cells (15 μM, 2 h incubation) by using confocal microscopy. Excitation was carried out with a blue light laser (λ_exc_ = 450 nm). As shown in Figure 9, a strong fluorescence signal was clearly observed inside cancer cells, thereby confirming an excellent and rapid cellular uptake. Worthy of note, no cell toxicity was appreciated during the experiments. The pattern of staining of **Ru4** and **Ru5** was similar and excluded nuclei as their preferential target. Indeed, the distribution of the red fluorescence emission of both compounds was found across the cytoplasm in punctate deposits, suggesting selective accumulation within intracellular vesicles. Important to note, it has been previously shown that the free ligand L5 was observed (λ_exc_ = 405 nm) evenly distributed around cell cytoplasm but not within nuclei of Hela cells, although only a partial correlation with MitoTracker Green (MTG) was observed.^50^

#### Dark Cytotoxicity

Ideally, the nonirradiated form of a given PACT compound should display low toxicity, while the irradiated form should exert biological activity.^64^ To assess the photoactivity of the present Ru(II) PACT compounds under biologically relevant conditions, we performed a screening in a panel of cell lines including both cancerous and noncancerous cells under dark conditions. For this screening, ovarian cancer (A2780), cervical cancer (HeLa), and melanoma (A375) cells along with nontumorigenic ovarian (CHO) cells were used; the clinical drug cisplatin was included for comparative purposes. As shown in Table 4, none of the complexes displayed antiproliferative activity after 48 h incubation, with negligible toxicity up to 100 μM, in contrast to cisplatin, which exerted cytotoxic activity against both normal and cancer cells in the low micromolar range. Importantly, no cell toxicity was found in normal CHO cells, which is an important requirement of anticancer drug development. Overall, these results validated our compounds as potential PACT agents since minimal dark toxicity is highly desirable in anticancer phototherapy.

**Table 4:** IC_50_ Values (μM) of Complexes **Ru1–Ru5** and CDDP at 48 h in the Dark

compound	A2780	HeLa	A375	CHO
**Ru1**	>100	>100	>100	>100
**Ru2**	>100	>100	>100	>100
**Ru3**	>100	>100	>100	>100
**Ru4**	>100	>100	>100	>100
**Ru5**	>100	>100	>100	>100
**CDDP**	1.9 ± 0.1	21 ± 2		7.8 ± 0.4

#### Photoactivation Studies

Once demonstrated that the compounds were not active under dark conditions, their ability to become toxic to cancer cells was evaluated using low doses of a visible light trigger (LED source at 465 nm, 4 mW/cm^2^, 1 h). Two cancer cell lines, namely, HeLa and A375 cells, were employed to illustrate this effect, and the phototoxic index (PI), defined as the ratio of dark to light IC_50_ values, served as a measure of the photoactivity. Since the complexes were deemed as inactive up to 100 μM, we included higher concentrations both in the presence or absence of light trigger to better characterize their photoactivation. To our delight, no cell inhibition was found with any complex, which corroborates their suitability for PACT (Table 4). In the presence of light irradiation, all the complexes showed antiproliferative activity in normoxic conditions (21% O_2_) against cancer cells, with IC_50_ values within the same micromolar range (Table 5). In general, higher photoactivation was found in A375 cells than in HeLa cells (PI values ranging from >3.6 to >29.1 compared to >6.8 to >14.8, respectively). Compound **Ru4**, bearing the nitro moiety, exhibited the higher phototoxic action in both cancer cell lines (PI values of >26.6 in A375 and >14.8 in HeLa), followed by compound **Ru1**. Interestingly, complex **Ru3** was barely photoactivated upon visible light irradiation against these cancer cell lines (PI values of >6.8 in both cancer cell lines). Overall, these results indicate that photoactivation of Ru(II) complexes bearing benzothiazolyl-1,2,3-triazole BTAT chelators can be achieved inside cancer cells. In contrast, light treatment had very little effect on the viability of cells treated with BTAT ligands, which exhibited very low cytotoxicity in each of the cell lines tested after 1 h treatment followed by 1 h irradiation under blue light (IC_50_ > 100 μM, Table S8). While our study demonstrates the noncytotoxic nature of the ligands after a 2 h treatment, in a previous study^50^ we reported cytotoxic effects after a 48 h exposure in the absence of light. This difference could be due to the prolonged accumulation of the ligands within HeLa cells over 48 h compared to the 2 h duration in our study. In our current research, we have demonstrated that the ruthenium complexes facilitate the entry of the ligands into the cells, which subsequently become cytotoxic upon irradiation and breakdown under blue light irradiation.

**Table 5:** IC_50_ Values (μM) and Phototoxicity Index (P.I.) under Normoxia of **Ru1–Ru5**^a^

	HeLa			A375		
compound	dark	blue light	P.I_HeLa_	dark	blue light	P.I_A375_
**Ru1**	>500	48.0 ± 3.4	>10.4	>500	20.55 ± 1.5	>24.4
**Ru2**	>500	58.5 ± 4.6	>8.6	>500	17.2 ± 3.0	>29.1
**Ru3**	>500	73.5 ± 4.2	>6.8	>500	73.5 ± 4.3	>6.8
**Ru4**	>500	33.8 ± 5.5	>14.8	>500	18.8 ± 3.7	>26.6
**Ru5**	>500	41.8 ± 3.9	>12.0	>500	27.1 ± 6.2	>18.5

a Compounds were incubated with the cells 1 h and then irradiated with blue light (λ = 465 nm, 4 mW/cm^2^) for 1 h. The phototoxicity index (P.I.) was calculated as IC_50 dark_/IC_50 λ=465nm_.

We have also performed the phototoxicity test in both A375 and HeLa cell lines under hypoxic conditions. The results reveal a contrasting pattern, where the compounds exhibit partially greater toxicity under normoxia conditions compared to hypoxia (Table 6).

**Table 6:** IC_50_ Values (μM) and Phototoxicity Index (P.I.) under Hypoxia (2% O_2_) of the Compounds **Ru1**–**Ru5**^a^

	HeLa			A375		
compound	dark	blue light	P.I_HeLa_	dark	blue light	P.I_A375_
**Ru1**	>500	70.2 ± 5.0	>7.1	>500	66.9 ± 6.1	>7.5
**Ru2**	>500	46.0 ± 3.9	>10.9	>500	>100	
**Ru3**	>500	85.2 ± 4.4	>5.9	>500	>100	
**Ru4**	>500	41.7 ± 2.7	>12.0	>500	51.9 ± 2.4	>9.6
**Ru5**	>500	57.2 ± 6.4	>8.7	>500	>100	

a Compounds were incubated with the cells 1 h and then irradiated with blue light (λ = 465 nm, 4 mW/cm^2^) for 1 h. The phototoxicity index (P.I.) was calculated as IC_50 dark_/IC_50 λ=465nm_.

Lower phototoxicity of light-activated drugs in oxygen-deprived environments can be a sign either that the phototoxicity under normoxia involves some form of a photodynamic effect, or that the hypoxic cells are more difficult to kill than normoxic cells, as hypoxia triggers a range of resistance effects.^64,65^

**Figure 10:**
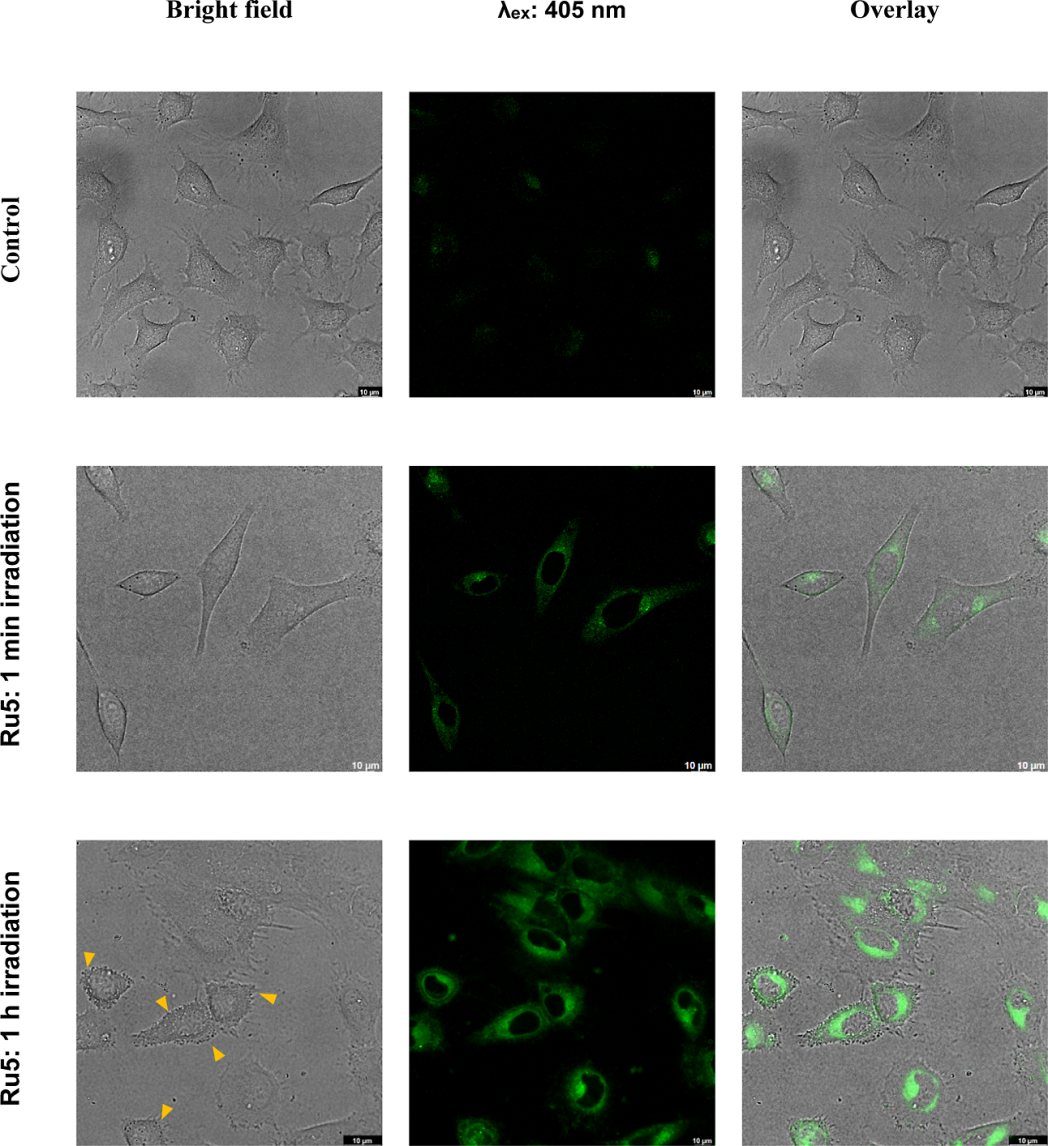
Confocal microscopy images of HeLa cells treated with **Ru5** at 15 μM for 1 h followed by blue light irradiation for 1 min and 1 h. λ_ex_: 405 nm, λ_em_: 516 ± 30 nm. The control group was maintained under untreated conditions. Scale bar: 10 μm. The orange triangles indicate the cellular membrane irregularities or blebs associated with apoptosis.

Confocal microscopy imaging of HeLa cells treated with **Ru5** under two different irradiation timings reveals distinct cellular responses. After 1 min of irradiation under blue light, no cell cytotoxicity was observed (Figure 10, first row, bright field image). In contrast, following 1 h irradiation, discernible cell damage is evident, characterized by the presence of cytoplasmic blebbing and notable morphology changes (Figure 10, orange triangles). These imaging data provide robust support for the photocytotoxicity results previously described, demonstrating the heightened cytotoxic effects of Ru BTAT compounds after blue light irradiation, as opposed to their relative dark condition or cells treated with the ligands alone.

The higher fluorescence signal observed in cells treated for 1 h under blue light, in comparison to 1 min of irradiation, is attributed mainly to the photoejection of the ligand after irradiation.^50^ This comprehensive examination through confocal microscopy provides valuable insights into the photoresponsive behavior of **Ru5** and its impact on cellular structures, further reinforcing its potential as an effective photoactive agent in phototherapy applications.

## Conclusions

This work shows the electronic, physicochemical, and biological properties of a new series of Ru(II) heteroleptic photocages [Ru(phen)_2_(BTAT)]^2+^ containing a novel type of minimal strained N,N-chelator, based on substituted 2-(1-(aryl)-1,2,3-triazol-4-yl)benzothiazoles possessing a push–pull architecture. The crystal structures of **Ru1**, **Ru2**, and **Ru5** showed the quasi-planar coordination of the BTAT ligands. Upon irradiation in water with blue light (λ_ex_ = 465 nm, 4 mW/cm^2^) photoejection of the ligand BTAT was observed by HPLC-MS spectrometry and UV–vis spectroscopy, with *t*_1/2_ ranging from 4.5 to 14.15 min depending of the electronic properties of the corresponding BTAT, being the one containing the more electron withdrawing substituent, **Ru4**, the less photolabile. A DFT mechanism for the photoejection of the BTAT ligand from Ru the complexes has been proposed. The new complexes showed very low toxicity in the dark even at high concentrations against the human cancer cells A375, HeLa, and A2780, and nontumorigenic ovarian CHO cells. They showed high phototoxicity toward cancer cells by blue light irradiation with high phototoxicity indexes in both normoxic and hypoxic conditions. An enhancement of the emission intensity of HeLa cells treated with **Ru5** was observed in response to increasing doses of light confirming the photoejection of the BTAT ligand. These studies suggest that BTAT could serve as a photocleavable protecting group for the cytotoxic bis-aqua ruthenium warhead [Ru(phen)_2_(OH_2_)_2_]^2+^.

## Experimental Section

### Materials

Potassium trifluoromethanesulfonate and 1,10-phenanthroline monohydrate were acquired from Sigma-Aldrich (Merk, Spain) and ruthenium trichloride trihydrate (RuCl_3_·3H_2_O) from Johnson Matthey. Deuterated solvents were obtained from Eurisotop, except for deuterated trifluoroacetic acid, which was obtained from Sigma-Aldrich. All chemicals were used as received without further purification.

### Characterization Techniques

^1^H NMR and ^13^C NMR experiments have been recorded on Bruker AV 400 or Buker AV 600 spectrometers, and ^19^F NMR experiments were carried out on a Bruker AV200 spectrometer. The ^1^H NMR and ^13^C NMR chemical shifts have been referenced to tetramethylsilane and were determined by referencing the residual ^1^H and ^13^C signal of the deuterated solvent. The ^19^F NMR chemical shifts have been referenced to the deuterated trifluoroacetic acid signal. UV–visible absorption spectra were recorded on a PerkinElmer Lambda 750 S spectrometer. Emission spectra were recorded on a Jobin Yvon Fluorolog 3–22 spectrofluorometer with a 450 W xenon lamp, two double-slit monochromators and a TBX-04 photomultiplier. Measurements were made in a fluorescence quartz cuvette with a 10 mm optical path. The lifetimes of the excited state were determined using an IBH FuoroHub TCSPC controller and a NanoLED pulse diode as the driving source, with an estimated measurement error of ±10% or better. Emission quantum yields (Φ) were measured using a Hamamatsu C11347 Absolute PL Quantum Yield spectrometer; the estimated uncertainty is ±5% or better. For measurements in deaerated conditions, solutions of the samples were previously degassed by bubbling argon for 30 min. ESI-MS spectra (positive mode) were recorded on an Agilent 6220 HPLC-MS TOF or an Agilent 1290 Series II HPLC coupled to an Agilent 6550 i-Funnel Q-TOF MS. HPLC experiments were carried out in a VWR-Hitachi, Elite LaChrom model, with a DAD detector, using a Teknokroma C18 chromatography column (25 × 0.46 cm, 5 μm). FT-IR spectra were recorded on a Jasco FT-IR-4600 spectrometer with a bounce-only ATR-PRO ONE system with a monolithic diamond prism. Elemental analysis experiments for carbon, hydrogen, nitrogen, and sulfur were performed on a LECO CNHS-932 microanalyzer. Reactions were carried out in 10 mL glass reaction vials in an Anton Paar Monowave 50 microwave (315 W).

### General Procedure of Synthesis of Ru(II) Complexes

In a glass reaction vial, 6 mL of 1:1 (v/v) EtOH/H_2_O mixture was poured and Ru(phen)_2_Cl_2_·2H_2_O (0.2 mmol),^66^ the respective BTAT ligand (**L1–L5**) (1 equiv, 0.2 mmol) and potassium triflate (3 equiv, 0.6 mmol) were added. The suspension is microwaved and heated at 120 °C for 2 min. The residue is taken to dryness, after that it was dissolved in dichloromethane and filtered to eliminate the salts. The solvent was then removed and the residue was purified by column chromatography on alumina using an ACN/DCM/MeOH 7:2:1 (v/v/v) mixture as eluent. The solution was taken to dryness and the residue was dissolved in dichloromethane and precipitated with hexane. The solid was filtered, washed with ethyl ether (2 × 5 mL), and dried in vacuo.

#### [Ru(phen)_2_(L1)][OTf]_2_ (**Ru1**)

The compound was isolated as an orange solid. Yield 79%. ^1^H-RMN (400 MHz, DMSO-*d*_6_, δ): 10.61 (s, 1H), 8.88 (dd, *J* = 8.4, 3.2, 1.2 Hz, 2H), 8.8 (dd, *J* = 8.3 Hz, 1H), 8.75 (d, *J* = 8.3 Hz, 1H), 8.62 (d, *J* = 5.3 Hz, 1H), 8.50 (dd, *J* = 5.3, 1.3 Hz, 1H), 8.45–8.34 (m, 5H), 8.32 (d, *J* = 5.4 Hz, 1H), 7.98 (d, *J* = 5.3 Hz, 1H), 7.97–7.91 (m, 2H), 7.85 (dd, *J* = 8.3, 5.3 Hz, 1H), 7.69 (dd, *J* = 8.3, 5.3 Hz, 1H), 7.61–7.53 (d, *J* = 8.2 Hz, 2H), 7.45 (dd, *J* = 7.6, 1.3 Hz, 1H), 7.37 (d, *J* = 8.2 Hz, 2H), 7.10 (dd, *J* = 7.6, 1.3 Hz, 1H), 5.90 (d, *J* = 8.5 Hz, 1H), 2.33 (s, 3H). ^13^C RMN (101 MHz, DMSO-*d*_6_, δ): 159.4(q), 154.3, 153.9, 153.4, 153.3, 150.7(q), 148.1(q), 147.8(q), 147.4(q), 147.3(q), 144.4(q), 140.1(q), 137.3, 137.2, 137.0, 134.2(q), 133.4(q), 130.5(q), 130.4, 130.0(q), 128.3, 128.2, 128.0, 127.9, 127.0, 126.8, 126.7, 126.6, 126.4, 125.8, 125.1, 120.3, 118.2, 20.7. TOF-HRMS (ESI+) *m*/*z*: [M]^2+^ calcd for C_40_H_28_N_8_RuS, 377.0595; found, 377.0596. Anal. Calcd for C_42_H_28_F_6_N_8_O_6_RuS_3_: C, 47.95; H, 2.68; N, 10.65; S, 9.14. Found: C, 47.84; H, 2.73; N, 10.65; S, 9.07.

#### [Ru(phen)_2_(L2)][OTf]_2_ (**Ru2**)

The compound was isolated as an orange solid. Yield 70%. ^1^H-RMN (400 MHz, DMSO-*d*_6_, δ): 10.68 (s, 1H), 8.89 (ddd, *J* = 8.3, 2.8, 1.3 Hz, 2H), 8.84 (dd, *J* = 8.3, 1.3 Hz, 1H), 8.75 (dd, *J* = 8.4, 1.3 Hz, 1H), 8.64 (dd, *J* = 5.2, 1.2 Hz, 1H), 8.50 (dd, *J* = 5.2, 1.3 Hz, 1H), 8.45–8.34 (m, 5H), 8.32 (dd, *J* = 5.3, 1.3 Hz, 1H), 8.00–7.92 (m, 3H), 7.86 (dd, *J* = 8.1, 5.3 Hz, 1H), 7.80–7.72 (m, 2H), 7.69 (dd, *J* = 8.1, 5.3 Hz, 1H) 7.49–7.41 (m, 3H), 7.10 (dd, 8.2, 1.3 Hz, 1H), 5.91 (d, *J* = 8.4 Hz, 1H). ^13^C RMN (101 MHz, DMSO-*d*_6_, δ): 162.3 (d, *J* = 247.7 Hz), 159.3(q), 154.2, 153.8, 153.3, 153.3, 150.7(q), 148.1(q), 147.7(q), 147.3(q), 147.3(q), 144.4(q), 137.3, 137.1, 137.0, 134.2(q), 132.2(q), 130.4(q), 130.4(q), 130.3(q), 130.0(q), 128.3, 128.2, 128.0, 127.9, 127.0, 126.7, 126.6, 126.4, 125.8, 125.0, 123.1 (d, *J* = 9.3 Hz), 118.2, 116.9 (d, *J* = 23.6 Hz). ^19^F RMN (188 MHz, DMSO-*d*_6_, δ): −76.40 (s), −109.21 (tt, *J* = 8.5, 4.1 Hz). TOF-HRMS (ESI+) *m*/*z*: [M]^2+^ calcd for C_39_H_25_FN_8_RuS, 379.0470; found, 379.0453 (*m*/*z*). Anal. Calcd for C_41_H_25_F_7_N_8_O_6_RuS_3_: C, 46.64; H, 2.39; N, 10.61; S, 9.11. Found: C 46.41; H, 2.44; N, 10.62; S, 9.06.

#### [Ru(phen)_2_(L3)][OTf]_2_ (**Ru3**)

The compound was isolated as an orange solid. Yield 71%. ^1^H-RMN (400 MHz, DMSO-*d*_6_, δ): 10.89 (s, 1H), 8.89 (ddd, *J* = 8.4, 3.5, 1.3 Hz, 2H), 8.85 (dd, *J* = 8.3, 1.3 Hz, 1H), 8.77 (dd, *J* = 8.3, 1.3 Hz, 1H), 8.67 (dd, *J* = 5.3, 1.3 Hz, 1H), 8.51 (dd, *J* = 5.2, 1.3 Hz, 1H), 8.46–8.34 (m, 5H), 8.32 (dd, *J* = 5.4, 1.3 Hz, 1H), 8.04–7.97 (m, 3H), 7.97–7.91 (m, 4H), 7.86 (dd, *J* = 8.2, 5.3 Hz, 1H), 7.70 (dd, *J* = 8.3, 5.3 Hz, 1H), 7.47 (dd, *J* = 8.4, 7.3, 1.1 Hz, 1H), 7.11 (ddd, *J* = 8.5, 7.2, 1.3 Hz, 1H), 5.91 (d, *J* = 8.5 Hz, 1H). ^13^C RMN (101 MHz, DMSO-*d*_6_, δ): 159.2(q), 154.3, 153.9, 153.3, 150.7(q), 148.1(q), 147.7(q), 147.3(q), 144.7(q), 138.4(q), 137.4, 137.2, 137.1, 134.3(q), 130.5(q), 130.4(q), 130.4(q), 130.0(q), 129.9 (q, *J* = 33.3 Hz), 128.3, 128.2, 128.0, 127.9, 127.4, 127.4, 127.2, 127.1, 126.8, 126.7, 126.4, 125.9, 125.1, 123.6 (q, *J* = 272.9 Hz), 121.1, 118.3. ^19^F RMN (188 MHz, DMSO-*d*_6_, δ): −59.86 (s), −76.40 (s). TOF-HRMS (ESI^+^) *m*/*z*: [M]^2+^ calcd for C_40_H_25_F_3_N_8_RuS, 404.0453; found, 404.0434 (*m*/*z*). Anal. Calcd for C_42_H_25_F_9_N_8_O_6_RuS_3_: C, 45.61; H, 2.28; N, 10.13; S, 8.70. Found: C, 45.60; H, 2.29; N, 9.95; S, 8.72.

#### [Ru(phen)_2_(L4)][OTf]_2_ (**Ru4**)

The compound was isolated as an orange solid. Yield 68%. ^1^H RMN (600 MHz, DMSO-*d*_6_, δ): 10.59 (s, 1H), 8.86 (ddd, *J* = 8.3, 3.0, 1.2 Hz, 2H), 8.83 (dd, *J* = 8.3, 1.3 Hz, 1H), 8.75 (dd, *J* = 8.3, 1.2 Hz, 1H), 8.65 (dd, *J* = 5.2, 1.3 Hz, 1H), 8.51 (dd, *J* = 5.3, 1.3 Hz, 1H), 8.48–8.43 (m, 2H), 8.42–8.33 (m, 5H), 8.30 (dd, *J* = 5.3, 1.3 Hz, 1H), 7.98 (dd, *J* = 5.3, 1.3 Hz, 1H), 7.97–7.91 (m, 4H), 7.85 (dd, *J* = 8.3, 5.3 Hz, 1H), 7.69 (dd, *J* = 8.3, 5.3 Hz, 1H), 7.47 (ddd, *J* = 8.3, 7.2, 1.1 Hz, 1H), 7.11 (ddd, *J* = 8.5, 7.2, 1.3 Hz, 1H), 5.91 (d, *J* = 8.4 Hz, 1H). ^13^C RMN (101 MHz, DMSO-*d*_6_, δ): 159.1(q), 154.4, 154.0, 153.4, 150.7(q), 148.1(q), 147.7(q), 147.5(q), 147.4(q), 145.0(q), 139.7(q), 137.5, 137.4, 137.2, 134.4(q), 130.6(q), 130.5(q), 130.4(q), 130.4(q), 130.1(q), 128.4, 128.1, 128.0, 127.3, 127.3, 126.9, 126.7, 126.5, 126.0, 125.7, 125.1, 121.4, 118.3. TOF-HRMS (ESI^+^) *m*/*z*: [M]^2+^ calcd for C_39_H_25_N_9_O_2_RuS, 392.5442; found, 392.5452. Anal. Calcd for C_41_H_25_F_6_N_9_O_8_RuS_3_: C, 45.47; H, 2.33; N, 11.64; S, 8.88. Found: C, 45.30; H, 2.40; N, 11.38; S, 8.66.

#### [Ru(phen)_2_(L5)][OTf]_2_ (**Ru5**)

The compound was isolated as an orange solid. Yield 62%. ^1^H-RMN (600 MHz, DMSO-*d*_6_, δ): 10.18 (s, 1H), 8.85 (ddd, *J* = 8.3, 2.6, 1.2 Hz, 2H), 8.81 (dd, *J* = 8.3, 1.3 Hz, 1H), 8.72 (dd, *J* = 8.3, 1.2 Hz, 1H), 8.57 (dd, *J* = 5.3, 1.3 Hz, 1H), 8.49 (dd, *J* = 5.3, 1.3 Hz, 1H), 8.42–8.37 (m, 3H), 8.37–8.32 (m, 3H), 7.97 (dd, *J* = 5.3, 1.3 Hz, 1H), 7.94 (ddd, *J* = 8.3, 5.3, 1.9 Hz, 2H), 7.84 (dd, *J* = 8.3, 5.2 Hz, 1H), 7.67 (dd, *J* = 8.3, 5.3 Hz, 1H), 7.45 (ddd, *J* = 8.3, 7.2, 1.1 Hz, 1H), 7.43–7.39 (m, 2H), 7.09 (ddd, *J* = 8.5, 7.2, 1.2 Hz, 1H), 6.81–6.75 (m, 2H), 5.91 (d, *J* = 8.5 Hz, 1H), 2.93 (s, 6H). ^13^C RMN (101 MHz, DMSO-*d*_6_, δ): 159.4(q), 154.2, 153.8, 153.4, 153.3, 151.0(q), 150.7(q), 148.1(q), 147.8(q), 147.4(q), 147.3(q), 144.0(q), 137.2, 137.0, 136.8, 134.1(q), 130.4(q), 130.4(q), 130.3(q), 129.9(q), 128.2, 128.1, 127.9, 127.9, 126.9, 126.7, 126.3, 125.8, 125.6, 125.4, 124.9, 124.7(q), 121.4(q), 118.1, 111.9, 39.8. TOF-HRMS (ESI^+^) *m*/*z*: [M]^2+^ calcd for C_41_H_31_N_9_RuS, 391.5728; found, 391.5738. Anal. Calcd for C_43_H_31_F_6_N_9_O_6_RuS_3_: C, 47.78; H, 2.89; N, 11.66; S, 8.90. Found: C, 46.43; H, 2.96; N, 11.10; S, 8.78.

### X-ray Structure Determinations

Crystals suitable for X-ray diffraction of **Ru1**, **Ru2**, and **Ru5** were obtained by diffusion of Et_2_O into a diluted acetonitrile solution of complex **Ru1** and **Ru5** and in diluted EtOH solution of complex **Ru2**, respectively. Intensities were registered at low temperature on a Bruker D8QUEST diffractometer using monochromated Mo Kα radiation (λ = 0.71073 Å). Absorption corrections were based on multiscans (program SADABS).^67^ Structures were refined anisotropically using SHELXL-2018.^68^ Hydrogen atoms were included using rigid methyl groups or a riding model. A summary of crystal data collection and refinement parameters are given in Tables S1. CCDC reference numbers are 2284059 for **Ru1**, 2284060 for **Ru5**, and 2284061 for **Ru2**.

### Special Features

**Ru1**: The structure contains poorly resolved regions of residual electron density; this could not be adequately modeled and so was “removed” using the program SQUEEZE,^69^ which is part of the PLATON system. The void volume per cell was 445 Å^3^, with a void electron count per cell of 105 in one void per unit cell. This could be consistent with the presence of 1 acetonitrile per unit cell which accounts for 88 electrons per unit cell because of this electron difference the additional solvent was not taken account of when calculating derived parameters such as the formula weight because the nature of the solvent was not certain. In this structure, one triflate anion is disordered over two positions, ca. 52:48%. **Ru2**: the OH hydrogen atom on the two ethanol molecules were found from a difference map and were refined with SADI restrain. **Ru5**: the structure contains poorly resolved regions of residual electron density; this could not be adequately modeled and so was “removed” using the program SQUEEZE, which is part of the PLATON system. The void volume per cell was 382 Å^3^, with a void electron count per cell of 96. This additional solvent was not taken account of when calculating derived parameters such as the formula weight, because the nature of the solvent was uncertain. The solvent could be consistent with the presence of one Et_2_O per unit cell which just accounts for 84 electrons per unit cell. One triflate anion is disordered over two positions, ca. 66:34%.

### Solution Stability

The stability of complexes was evaluated by UV–vis spectra after 24 and 120 h at 37 °C. Complexes were dissolved in ACN or water at concentration 10 μM.

### Photoejection by UV–Vis

Photoejection experiments were carried out in duplicate in 10 mm quartz cuvettes on 3 mL solutions of 10 μM in H_2_O-mQ, and blue light irradiation λ = 465 nm, 4 mW/cm^2^. Irradiation intervals were as short as 60 s at early times and more than 150 s at later ones; experiments were considered complete when 150 s irradiation intervals produced no further discernible changes in the absorption spectrum. The normalized change in absorbance was plotted to determine the half-life of ejection using Graph Pad Prism 5.0 software as the published method by Glazer and co-workers.^60,70,71^ Half-life in this context refers to the time it takes to reach 1/2 of the maximum change in the signal used to monitor the process.

### Ligand Photoejection Experiments by HPLC-MS-TOF

Solutions 10^–4^ M in ACN of complexes **Ru1–Ru5** were prepared and divided into two aliquots: one aliquot was irradiated in a UV–vis spectroscopy cuvette with blue light (λ = 465 nm, 4 mW/cm^2^) for 60 min and the second aliquot was protected from light. The samples were analyzed in an Agilent 1290 series II HPLC equipment, with a DAD detector, coupled to an Agilent 6550 i-Funnel Q-TOF MS mass spectrometer. The column used is a C18 Zorbax Eclipse Plus column (10 × 2.1 cm, 1.8 μm). The method used uses as mobile phase: Milli-Q water (0.1% HCOOH) as phase A and ACN (0.1% HCOOH) as phase B. The flow is 0.4 mL/min in a gradient of: 0 min (99% A), 2 min (99% A), 22 min (100% B), 24 min (100% B), 25 min (99% A), 30 min (99% A).

### Singlet Oxygen Quantum Yield (Φ_Δ_)

Procurement was adapted from literature.^72,73^ Samples were prepared in an air-saturated acetonitrile solution 5 × 10^–6^ M. Absorbance of 1,3-diphenylisobenzofuran (DPBF) at 411 nm (5 × 10^–5^ M) was plotted against irradiation times (465 nm, 4 mW/cm^2^). Slope and linear regression were calculated. Singlet oxygen quantum yield where determined using the equation: , where Φ_Δr_ is the reference singlet oxygen quantum yield [Ru(bpy)_3_](PF_6_)_2_, Φ_Δ_ = 0.57 in aerated acetonitrile,^74^*m* are the slopes of samples and reference, and *A*λ are the absorbance of compounds and reference at irradiation wavelength.

### Phototoxicity Testing

For dark cytotoxic screening, A2780, CHO, HeLa, and A375 cells were cultured in 96-well plates at a density of 5 × 10^3^ cells/well in complete medium and incubated for 24 h at 310 K and 5% CO_2_ in a humidified incubator. Serial dilutions of tested compounds in cell culture media were then added at final concentrations in the range of 0 to 100 μM in a final volume of 100 μL/well (%v/v DMSO below 0.4%) for 48 h prior to MTT test. For photoactivation studies, HeLa and A375 cells were used. Treatments were added at final concentrations in the range of 0 to 500 μM. After 1 h incubation with the compounds, light irradiation treatments were applied using a LED photoreactor (Luzchem; Canada) fitted with LED lamps centered at 465 nm (final intensity 4 mW/cm^2^) for 1 h. Dark control analogues were directly kept in the dark for 2 h. After incubation periods, cells were washed with saline PBS buffer and loaded with 50 μL of MTT solution (1 mg/mL) for additional 4 h, then removed and 50 μL DMSO was added to solubilize the purple formazan crystals formed in active cells. The absorbance was measured at 570 nm using a microplate reader (FLUOstar Omega) and the IC_50_ values were calculated based on the inhibitory rate curves using the next the equation
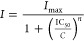
where *I* represents the percentage inhibition of viability observed, *I*_max_ is the maximal inhibitory effect, IC_50_ is the concentration that inhibits 50% of maximal growth, *C* is the concentration of the treatment, and *n* is the slope of the semilogarithmic dose–response sigmoidal curves. The nonlinear fitting was performed using SigmaPlot 14.0 software. Two independent experiments were performed with triplicate points per concentration level (*n* = 3).

### Confocal Microscopy

HeLa cells were seeded onto Ibidi μ-slides at 10^4^ cells/cm^2^ in a complete medium and incubated for 24 h at 310 K and 5% CO_2_ in a humidified incubator. Compounds were added at indicated concentrations for 2 h. Cells were then washed with PBS twice and imaged under confocal microscopy (STELLARIS Leica Microsystems) using 405 nm excitation laser.

### Computational Details

DFT calculations have been carried out on compounds **Ru1–Ru5** with full geometry optimization, by using the M06-L functional,^75^ the Lanl2tz(f) basis set^76,77^ for Ru and the 6-311G(d,p) basis set^78,79^ for the lighter atoms. The structure of the first triplet excited states, T_1_, was obtained by imposing an open shell triplet spin multiplicity. The solvents ACN and water were implicitly considered by using the PCM method.^80^ Frequency calculations, in the normal oscillator approximation, were carried out to check that the optimized geometries corresponded to energy minima on the potential energy surface. TD-DFT calculations have been performed using the same methods and models described above to calculate the electronic absorption spectra of the considered ruthenium compounds. All calculations have been performed by the Gaussian16 program package.^81^

## Supporting Information Available

Characterization of Ru(II) complexes, NMR spectra, ESI-MS, electronic spectra, HPLC, hydrogen bond and intermolecular π–π interactions in the crystal structures, and theoretical calculations (DOCX)
