# Supplementary material for: Photoactivatable Ruthenium Complexes Containing Minimal Straining Benzothiazolyl-1,2,3-triazole Chelators for Cancer Treatment
Source: Inorg Chem. 2024 Feb 22;63(14):6202–16. doi: 10.1021/acs.inorgchem.3c04432 (PMC11005040; doi:10.1021/acs.inorgchem.3c04432)
Supplement: Supplementary file 1 — ic3c04432_si_001.docx [file ic3c04432_si_001.docx]

**Supporting Information**

**Photoactivatable Ruthenium Complexes**

**Containing Minimal Straining Benzothiazolyl-1,2,3-Triazole (BTAT) Chelators for Cancer Treatment**

*Francisco J. Ballester ^a^, Alba Hernández ^a^, M. Dolores Santana ^a,*^, Delia Bautista ^b^, Pezhman Ashoo ^a^, Enrique Ortega-Forte ^a^, Giampaolo Barone ^c,*^, José Ruiz ^a,*^*

^a^ Departamento de Química Inorgánica and Regional Campus of International Excellence “Campus Mare Nostrum”, Universidad de Murcia, and Biomedical Research Institute of Murcia (IMIB-Arrixaca), E-30071 Murcia, Spain.

^b^ S.A.I., Universidad de Murcia, E-30071, Murcia, Spain.

^c^ Dipartimento di Scienze e Tecnologie Biologiche Chimiche e Farmaceutiche, Università degli Studi di Palermo, I-90128 Palermo, Italy.

Table of Contents

**Scheme S1**

**Figure S1.** ^1^H-NMR spectrum of compound **Ru1**, 400 MHz (DMSO-d_6_)

**Figure S2.** ^13^C-NMR spectrum of compound **Ru1**, 101 MHz (DMSO-d_6_)

**Figure S3.** DEPT-135 spectrum of compound **Ru1**, 101 MHz (DMSO-d_6_)

**Figure S4.** [^1^H-^1^H]-COSY spectrum of compound **Ru1**, 400 MHz (DMSO-d_6_)

**Figure S5**. [^1^H-^13^C]-HMQC spectrum of compound **Ru1**, 400 MHz (DMSO-d_6_)

**Figure S6.** [^1^H-^13^C]-HMBC spectrum of compound **Ru1**, 400 MHz (DMSO-d_6_)

**Figure S7.** [^1^H-^1^H]-NOESY spectrum of compound **Ru1**, 400 MHz (DMSO-d_6_)

**Figure S8.** ^1^H-NMR spectrum of compound **Ru2**, 400 MHz (DMSO-d_6_)

**Figure S9.** ^13^C-NMR spectrum of compound **Ru2**, 101 MHz (DMSO-d_6_)

**Figure S10.** DEPT-135 spectrum of compound **Ru2**, 101 MHz (DMSO-d_6_)

**Figure S11.** [^1^H-^1^H]-COSY spectrum of compound **Ru2**, 400 MHz (DMSO-d_6_)

**Figure S12**. ^19^F-{^1^H }-NMR spectrum of compound **Ru2**, 188 MHz (DMSO-d_6_)

**Figure S13**. [^1^H-^13^C]-HMQC spectrum of compound **Ru2**, 400 MHz (DMSO-d_6_)

**Figure S14.** [^1^H-^1^H]-NOESY spectrum of compound **Ru2**, 400 MHz (DMSO-d_6_)

**Figure S15.** ^1^H-NMR spectrum of compound **Ru3**, 400 MHz (DMSO-d_6_)

**Figure S16.** ^13^C-NMR spectrum of compound **Ru3**, 101 MHz (DMSO-d_6_)

**Figure S17.** DEPT-135 spectrum of compound **Ru3**, 101 MHz (DMSO-d_6_)

**Figure S18.** [^1^H-^1^H]-COSY spectrum of compound **Ru3**, 400 MHz (DMSO-d_6_)

**Figure S19**. ^19^F-{^1^H }-NMR spectrum of compound **Ru3**, 188 MHz (DMSO-d_6_).

**Figure S20**. [^1^H-^13^C]-HMQC spectrum of compound **Ru3**, 400 MHz (DMSO-d_6_)

**Figure S21.** [^1^H-^1^H]-NOESY spectrum of compound **Ru3**, 400 MHz (DMSO-d_6_)

**Figure S22.** ^1^H-NMR spectrum of compound **Ru4**, 600 MHz (DMSO-d_6_)

**Figure S23.** ^13^C-NMR spectrum of compound **Ru4**, 101 MHz (DMSO-d_6_)

**Figure S24.** DEPT-135 spectrum of compound **Ru4**, 101 MHz (DMSO-d_6_)

**Figure S25.** [^1^H-^1^H]-COSY spectrum of compound **Ru4**, 600 MHz (DMSO-d_6_)

**Figure S26**. [^1^H-^13^C]-HMQC spectrum of compound **Ru4**, 600 MHz (DMSO-d_6_).

**Figure S27.** ^1^H-NMR spectrum of compound **Ru5**, 600 MHz (DMSO-d_6_).

**Figure S28.** ^13^C-NMR spectrum of compound **Ru5**, 101 MHz (DMSO-d_6_)

**Figure S29.** DEPT-135 spectrum of compound **Ru5**, 101 MHz (DMSO-d_6_)

**Figure S30.** [^1^H-^1^H]-COSY spectrum of compound **Ru5**, 600 MHz (DMSO-d_6_)

**Figure S31**. [^1^H-^13^C]-HMQC spectrum of compound **Ru5**, 600 MHz (DMSO-d_6_)

**Figure S32.** [^1^H-^13^C]-HMBC spectrum of compound **Ru5**, 400 MHz (DMSO-d_6_)

**Figure S33.** ESI-MS of compound **Ru1**

**Figure S34.** ESI-MS of compound **Ru2**

**Figure S35.** ESI-MS of compound **Ru3**

**Figure S36.** ESI-MS of compound **Ru4**

**Figure S37.** ESI-MS of compound **Ru5**

**Figure S38.** Hydrogen bonds in the structure of **Ru1**. Ellipsoids have been represented at 50 % probability

**Figure S39.** Hydrogen bonds in the structure of **Ru2**. Ellipsoids have been represented at 50 % probability

**Figure S40**. Intermolecular π-π interactions involving the phen rings of **Ru1**

**Figure S41**. Intermolecular π-π interactions involving the phen rings of **Ru2**

**Figure S42**. Intermolecular π-π interactions involving the phen rings of **Ru5**

**Figure S43.** Absorptionspectra of complexes **Ru1- Ru5** in ACN.

**Figure S44.** Emissionspectra of complexes **Ru1-Ru5** in ACN.

**Figure S45.** Stability of **Ru1** (a), **Ru2** (b), **Ru3** (c), **Ru4** (d) and **Ru5 (e)** over 120 h in H_2_O at 310 K. t = 0 (grey), t = 24 h (blue) and t = 120 h (red line).

**Figure S46**. Changes in absorption spectra of **Ru2** (a), **Ru3** (b), **Ru4** (c) and **Ru5** (d) in H_2_O (10^-5^ M) as observed upon irradiation with blue ligth (λex = 465 nm, 3 mW/cm^2^) and photoejection kinetics.

**Figure S47**. Determination of photoejection products of **Ru1** by HPLC.

**Figure S48**. Determination of photoejection products of **Ru2** by HPLC.

**Figure S49**. Determination of photoejection products of **Ru3** by HPLC.

**Figure S50**. Determination of photoejection products of **Ru4** by HPLC.

**Figure S51**. Determination of photoejection products of **Ru5** by HPLC.

**Figure S52.** Structure of the Ru complexes **Ru1**- **Ru5** in water, singlet ground state, S_0_, obtained by DFT calculations.

**Figure S53.** Structure of the Ru complexes **Ru1**- **Ru5** in water, triplet excited state, T_1_, obtained by DFT calculations.

**Figure S54.** UV-visible absorption spectrum of the Ru complexes **Ru1** - **Ru5** in ACN, obtained by TD-DFT calculations.

**Figure S55.** Proposed mechanism of ligand photo-ejection for the Ru complex **Ru4** in H_2_O, obtained by DFT calculations.

**Table S1**. Crystal data and structure refinement for complexes **Ru1**, **Ru2** and **Ru5**.

**Table S2.** Hydrogen bonds for **Ru1** (Å and °).

**Table S3**. Hydrogen bonds for **Ru2** (Å and °).

**Table S4**. Selected π-π interaction parameters.

**Table S5**. Bond distance (Å) and angles (˚) between the Ru atom and the coordinated nitrogen atoms of BTAT ligand in the Ru(II) octahedral complexes **Ru1** - **Ru5**, obtained from the X-ray structures and from the calculated structures obtained by DFT calculations in water (H_2_O) in the singlet ground state, S_0_, and in the first triplet excited state, T_1_.

**Table S6.** Absorption wavelength (λ, nm), oscillator strength (f) and major contributions of occupied and virtual molecular orbitals (H: HOMO, L: LUMO), of the lower energy absorption transitions of compounds **Ru1** - **Ru5**, obtained by TD-DFT calculations in H_2_O solution.

**Table S7.** Dipole moments (Debye) of the Ru(II) octahedral complexes **Ru1** - **Ru5** in the ground state (μS_0_) and in the first triplet excited state (μT_1_), and their difference (μT_1_-μS_0_), obtained by DFT and TD-DFT calculations, in water (H_2_O) and in acetonitrile (ACN).

**Table S8**. (Photo)toxicity test (normoxia/O_2_ 21%) of ligands **L1**- **L5**: [1 h treatment + 1 h irradiation under blue light].

**Table S9**. Resonances of protons and carbons of the BTAT ligands

**Scheme S*1*.** Synthetic route for the preparation of 1,2,3-triazole derivatives. i) 5% CF_3_COOH, EtOH, 24 h, r.t. ii) DDQ, DCM anh., 2 h. 0 °C. ^1^

**Figure S1.** ^1^H-NMR spectrum of compound **Ru1**, 400 MHz (DMSO-d_6_).

**Figure S2.** ^13^C-NMR spectrum of compound **Ru1**, 101 MHz (DMSO-d_6_).

**Figure S3.** DEPT-135 spectrum of compound **Ru1**, 101 MHz (DMSO-d_6_).

**Figure S4.** [^1^H-^1^H]-COSY spectrum of compound **Ru1**, 400 MHz (DMSO-d_6_).

**Figure S5**. [^1^H-^13^C]-HMQC spectrum of compound **Ru1**, 400 MHz (DMSO-d_6_).

**Figure S6.** [^1^H-^13^C]-HMBC spectrum of compound **Ru1**, 400 MHz (DMSO-d_6_).


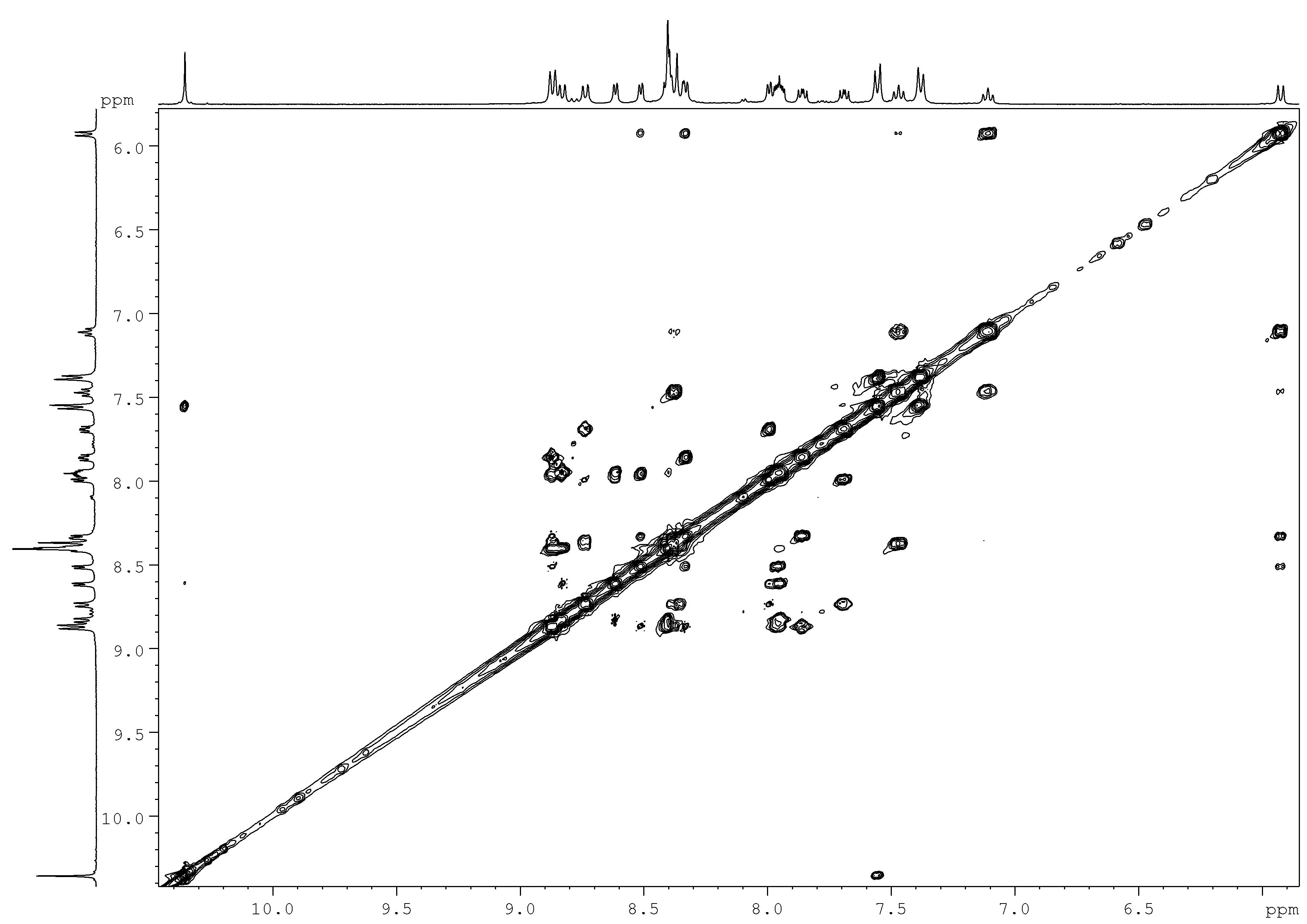


**Figure S7.** [^1^H-^1^H]-NOESY spectrum of compound **Ru1**, 400 MHz (DMSO-d_6_).

**Figure S8.** ^1^H-NMR spectrum of compound **Ru2**, 400 MHz (DMSO-d_6_).

**Figure S9.** ^13^C-NMR spectrum of compound **Ru2**, 101 MHz (DMSO-d_6_).

**Figure S10.** DEPT-135 spectrum of compound **Ru2**, 101 MHz (DMSO-d_6_).

**Figure S11.** [^1^H-^1^H]-COSY spectrum of compound **Ru2**, 400 MHz (DMSO-d_6_).

**Figure S12**. ^19^F-{^1^H }-NMR spectrum of compound **Ru2**, 188 MHz (DMSO-d_6_).

**Figure S13**. [^1^H-^13^C]-HMQC spectrum of compound **Ru2**, 400 MHz (DMSO-d_6_).

**
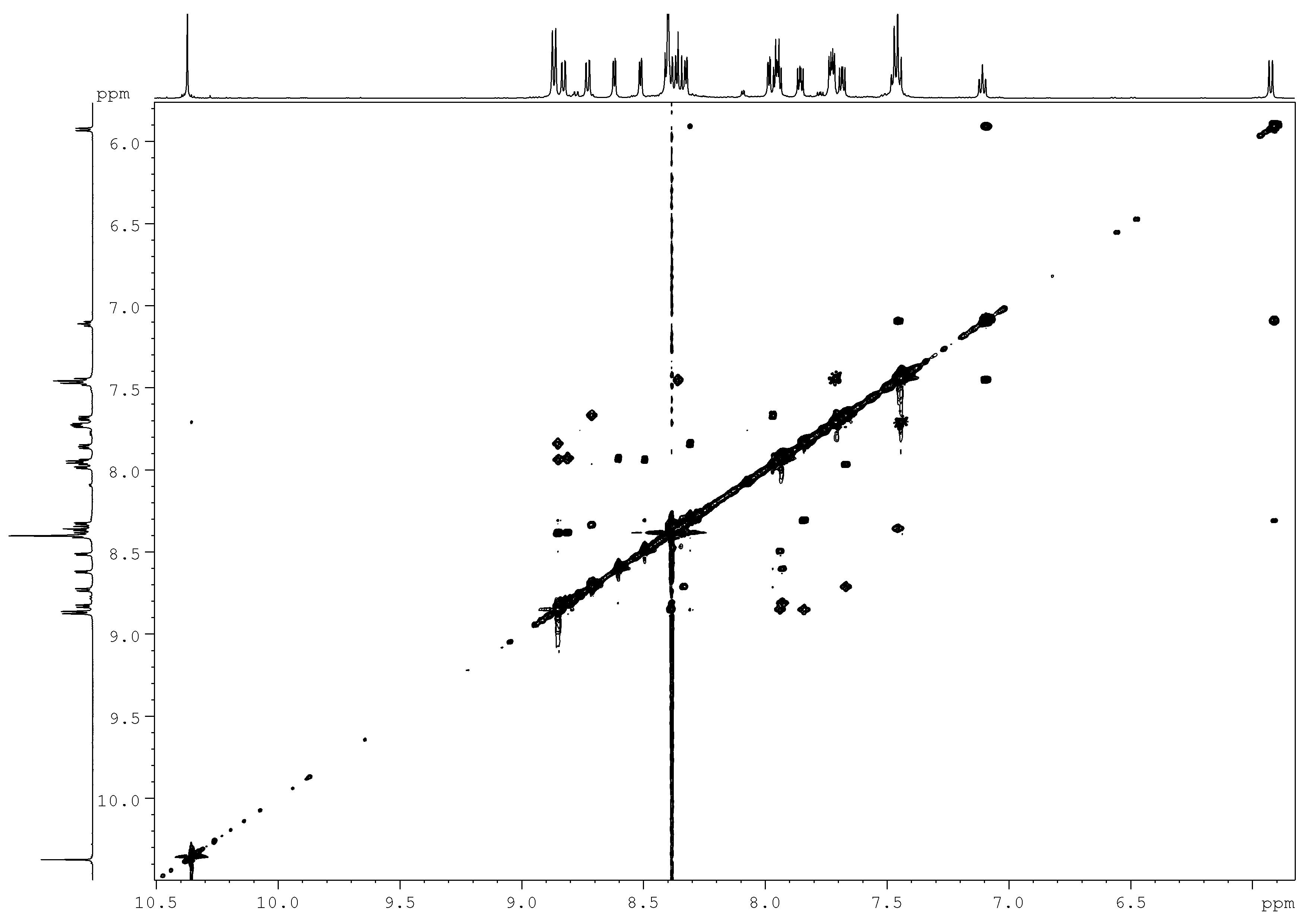
**

**Figure S14**. [^1^H-^1^H]-NOESY spectrum of compound **Ru2**, 400 MHz (DMSO-d_6_).

**Figure S15.** ^1^H-NMR spectrum of compound **Ru3**, 400 MHz (DMSO-d_6_).

**Figure S16.** ^13^C-NMR spectrum of compound **Ru3**, 101 MHz (DMSO-d_6_).

**Figure S17.** DEPT-135 spectrum of compound **Ru3**, 101 MHz (DMSO-d_6_).

**Figure S18.** [^1^H-^1^H]-COSY spectrum of compound **Ru3**, 400 MHz (DMSO-d_6_).

**Figure 19**. ^19^F-{^1^H }-NMR spectrum of compound **Ru3**, 188 MHz) (DMSO-d_6_).

**Figure S20**. [^1^H-^13^C]-HMQC spectrum of compound **Ru3**, 400 MHz (DMSO-d_6_).


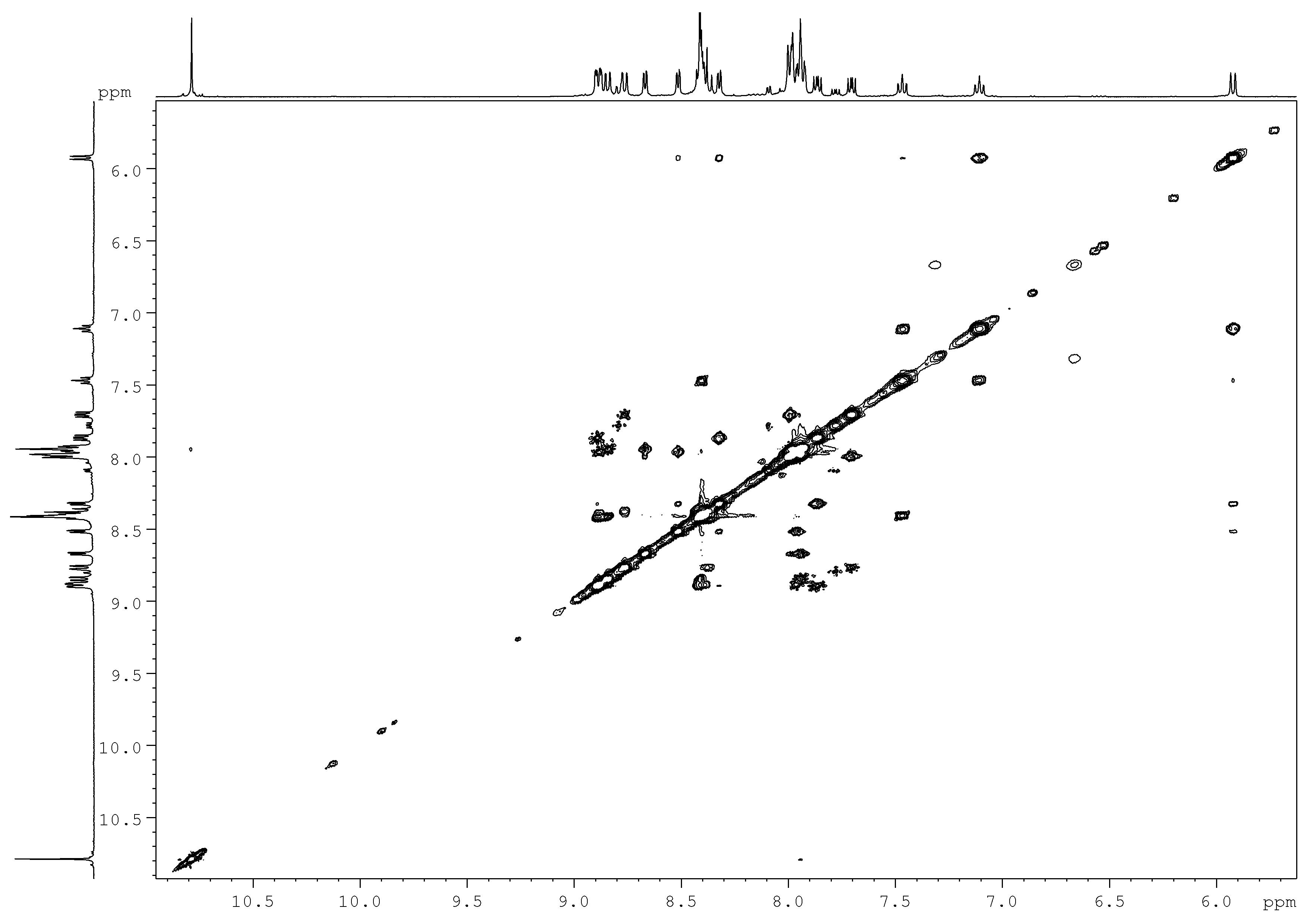


**Figure S21**. [^1^H-^1^H]-NOESY spectrum of compound **Ru3**, 400 MHz (DMSO-d_6_).

**Figure S22.** ^1^H-NMR spectrum of compound **Ru4**, 600 MHz (DMSO-d_6_)

**Figure S23.** ^13^C-NMR spectrum of compound **Ru4**, 101 MHz (DMSO-d_6_).

**Figure S24.** DEPT-135 spectrum of compound **Ru4**, 101 MHz (DMSO-d_6_).

**Figure S25.** [^1^H-^1^H]-COSY spectrum of compound **Ru4**, 600 MHz (DMSO-d_6_).

**Figure S26**. [^1^H-^13^C]-HMQC spectrum of compound **Ru4**, 600 MHz (DMSO-d_6_).

**Figure S27.** ^1^H-NMR spectrum of compound **Ru5**, 600 MHz (DMSO-d_6_).

**Figure S28.** ^13^C-NMR spectrum of compound **Ru5**, 151 MHz (DMSO-d_6_).

**Figure S29.** DEPT-135 spectrum of compound **Ru5**, 151 MHz (DMSO-d_6_).

**Figure S30.** [^1^H-^1^H]-COSY spectrum of compound **Ru5**, 600 MHz (DMSO-d_6_).

**Figure S31**. [^1^H-^13^C]-HMQC spectrum of compound **Ru5**, 600 MHz (DMSO-d_6_).

**Figure S32.** [^1^H-^13^C]-HMBC spectrum of compound **Ru5**, 400 MHz (DMSO-d_6_).

**Figure S33.** ESI-MS of compound **Ru1**.

**Figure S34**. ESI-MS of compound **Ru2**.

**Figure S35**. ESI-MS of compound **Ru3**.

**Figure S36.** ESI-MS of compound **Ru4**. The 922.0120 peak is an internal reference.

**Figure S37.** ESI-MS of compound **Ru5**.


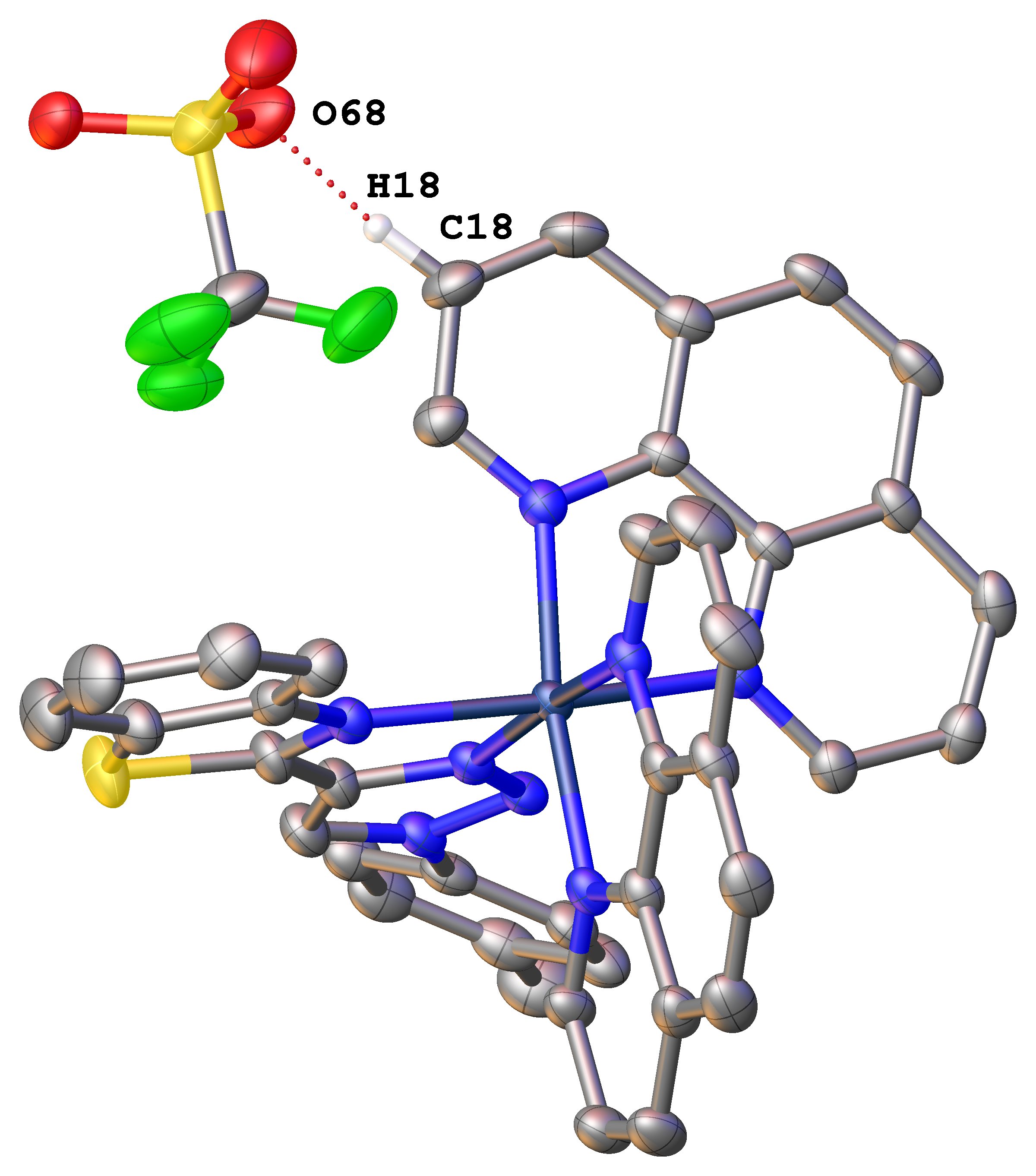


**Figure S38.** Hydrogen bonds in the structure of **Ru1**. Ellipsoids have been represented at 50 % probability.


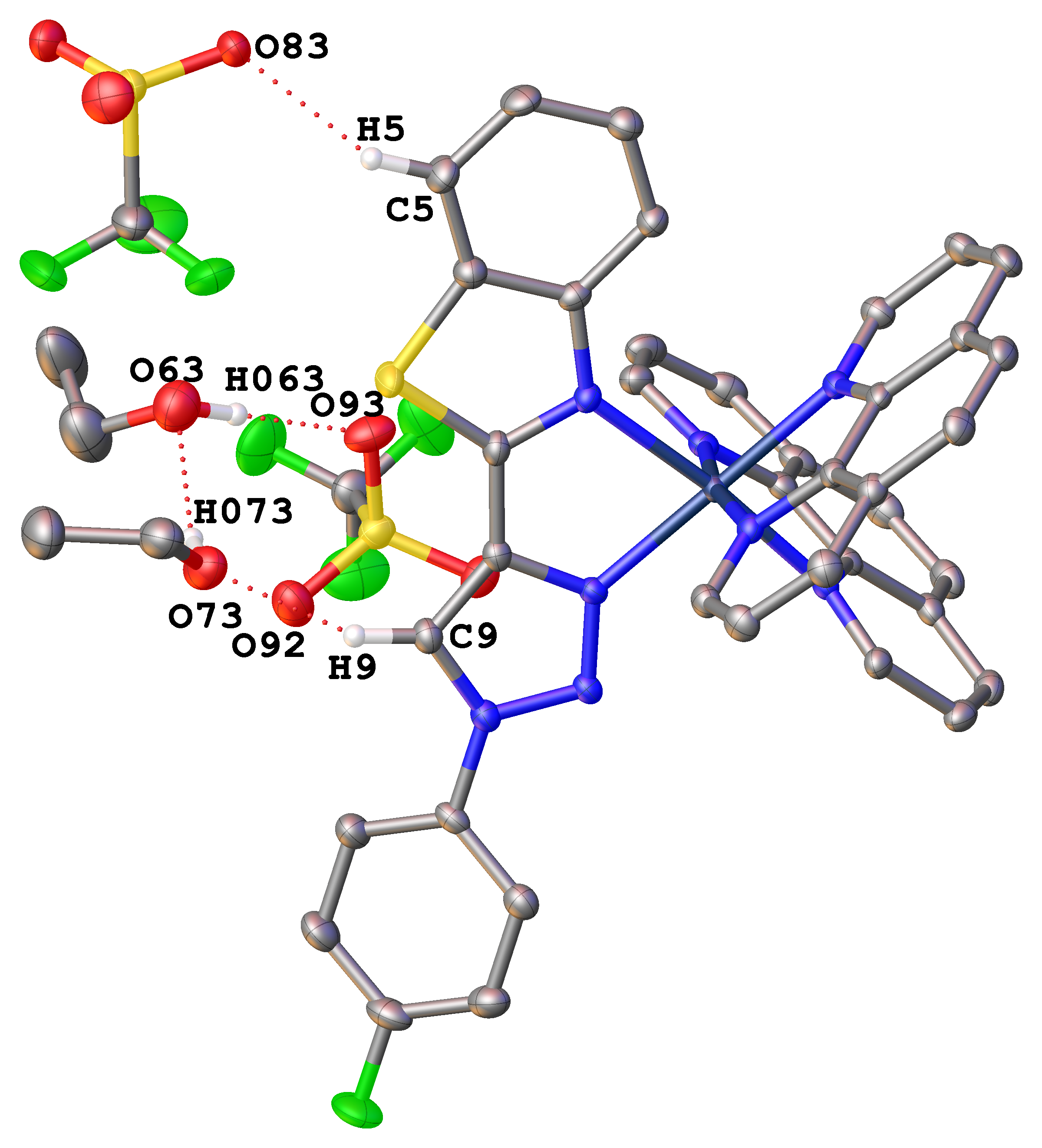


**Figure S39.** Hydrogen bonds in the structure of **Ru2**. Ellipsoids have been represented at 50 % probability.


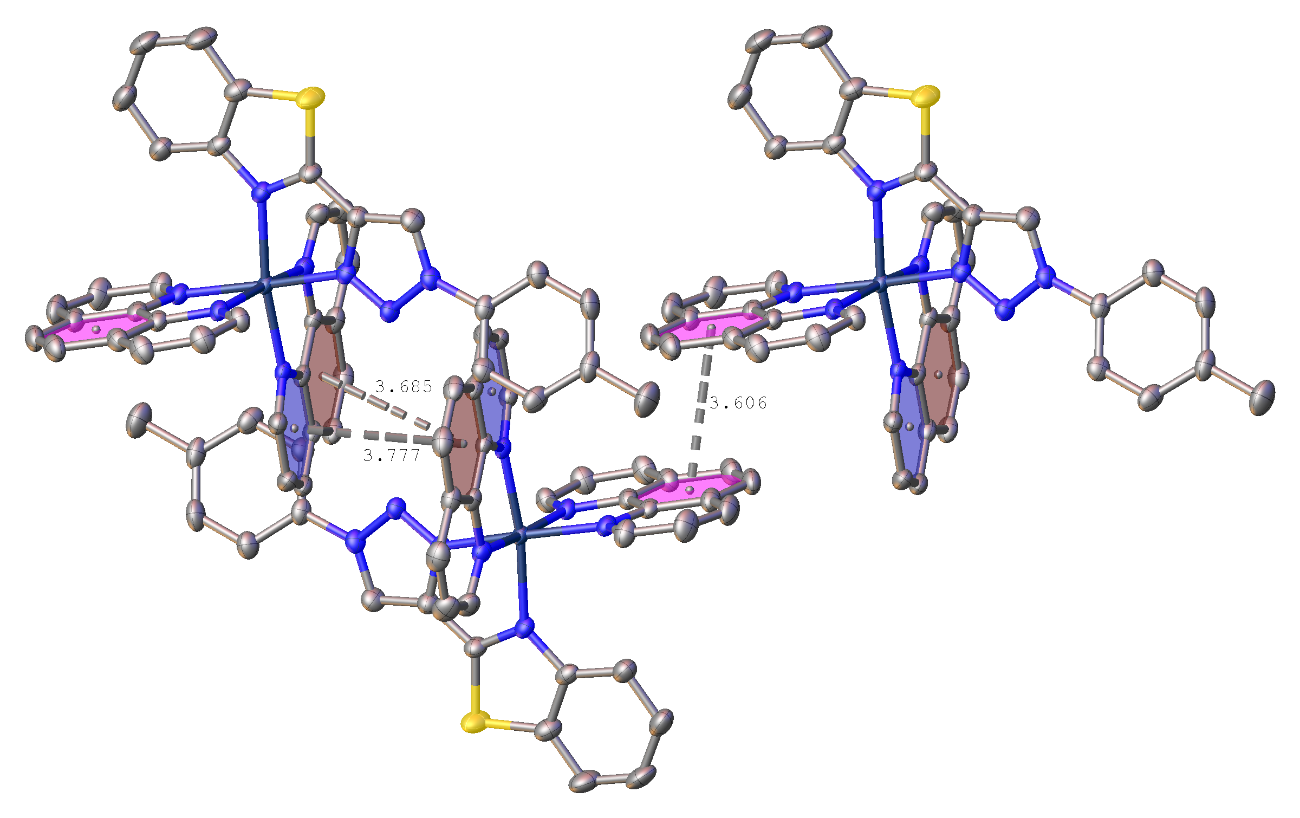


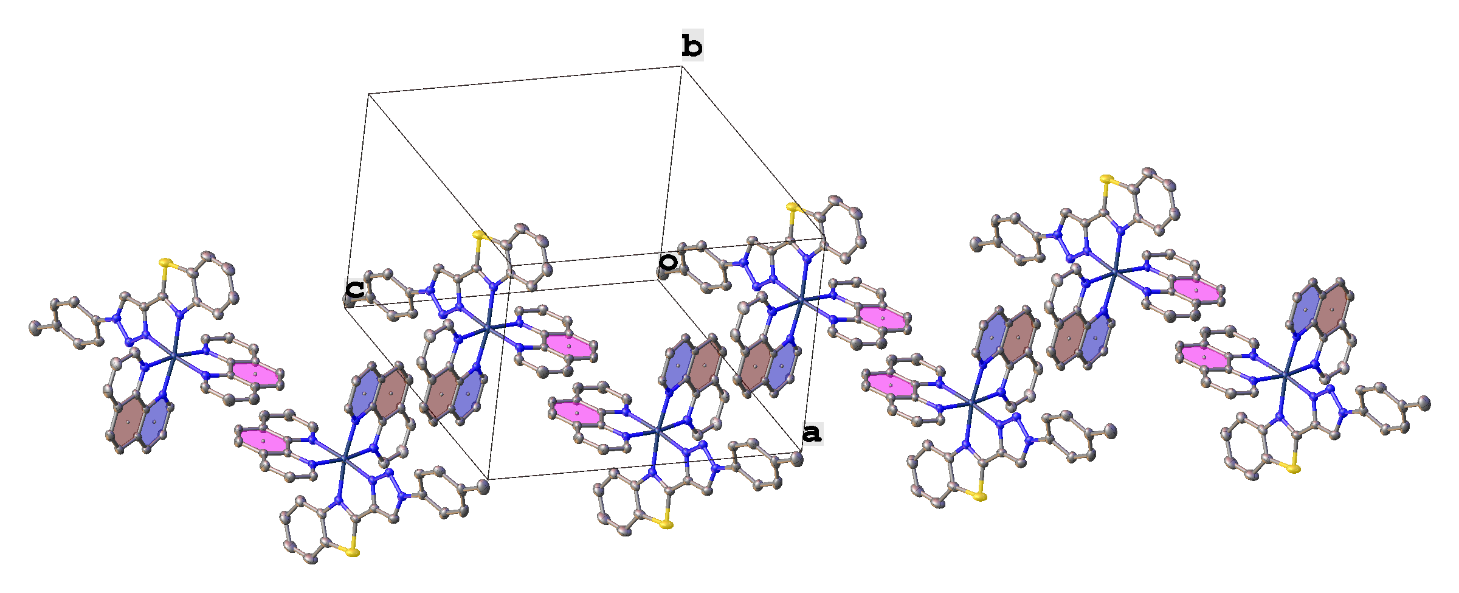


**Figure S40**. Intermolecular π-π interactions involving the phen rings of **Ru1** andthe chains along c axis.


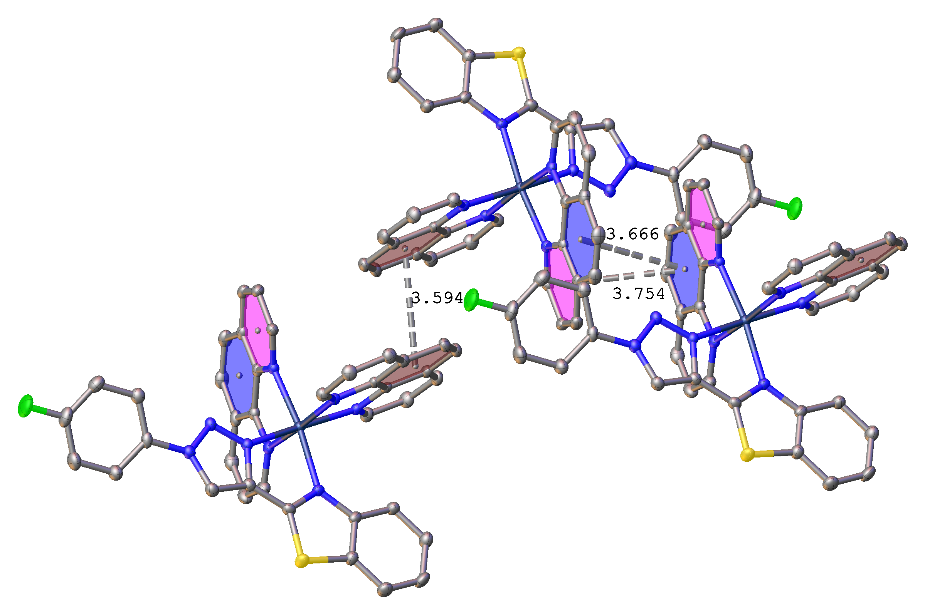


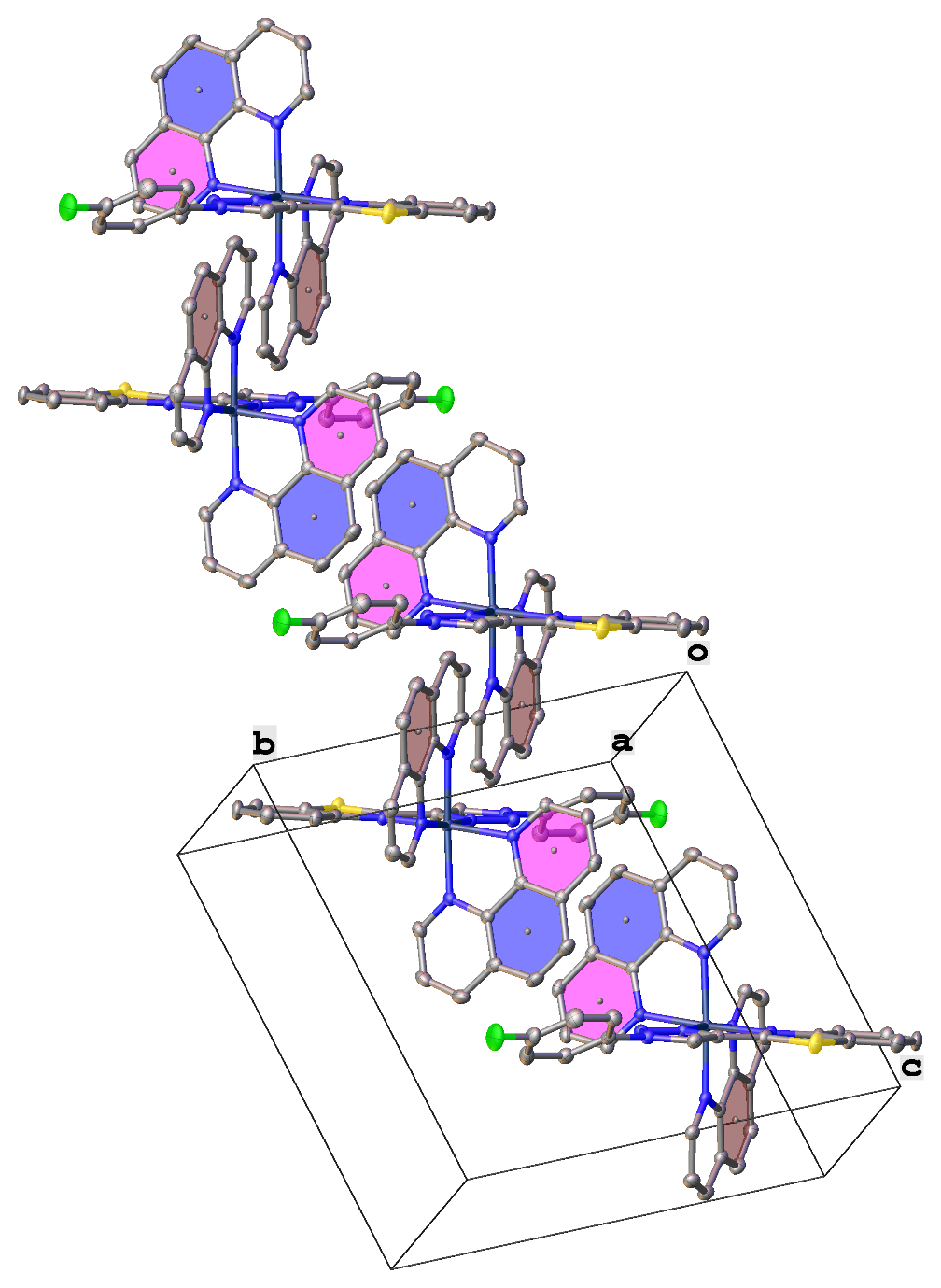


**Figure S41**. Intermolecular π-π interactions involving the central phen of **Ru2** andthe chains along c axis.


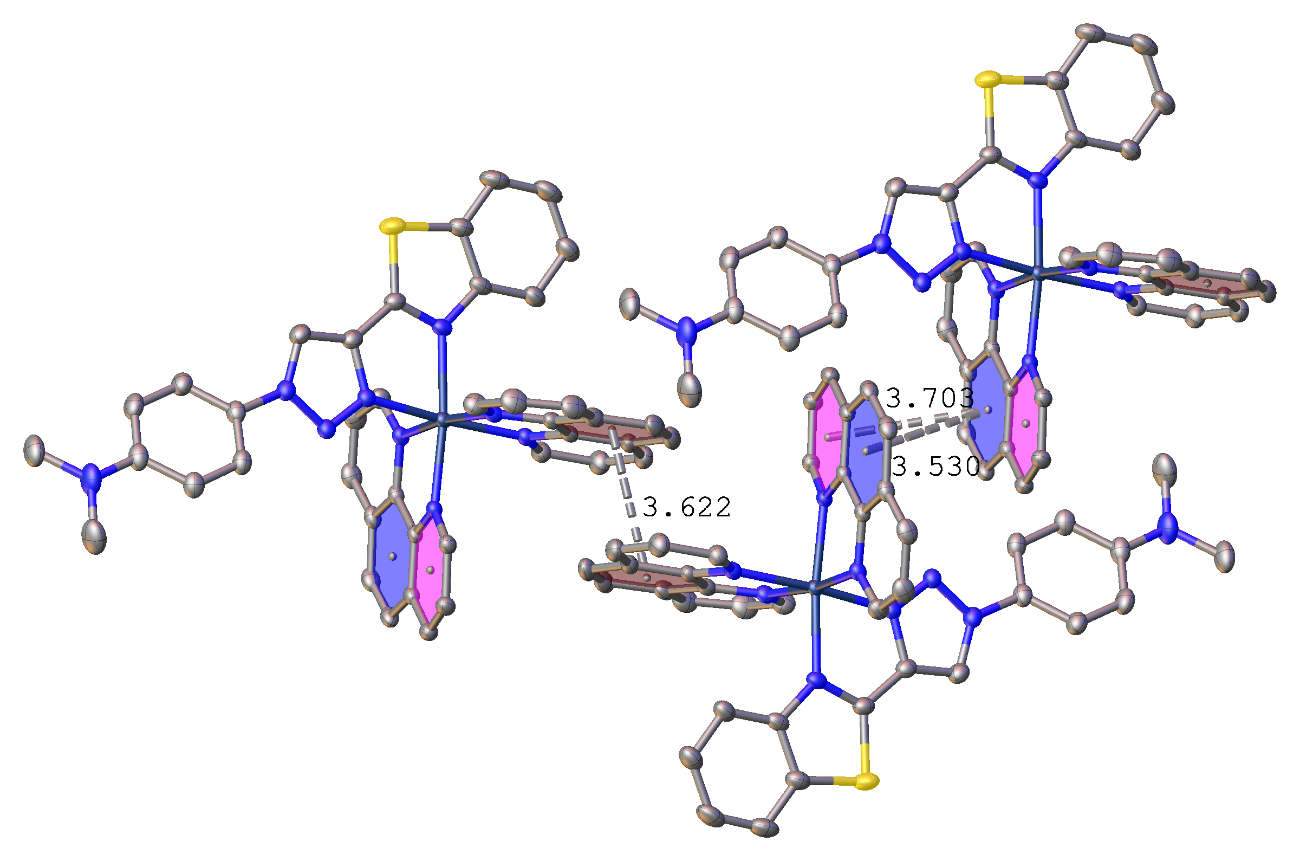


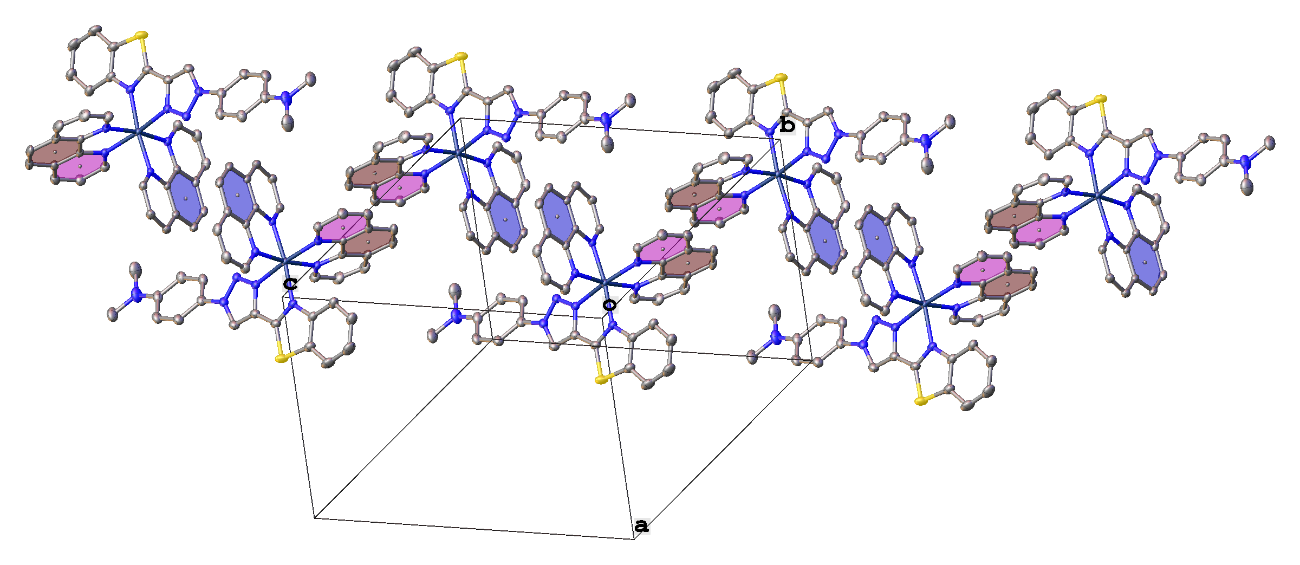


**Figure S42**. Intermolecular π-π interactions involving the central phen of **Ru5** andthe chains along c axis.

**Figure S43.** Absorptionspectra of complexes **Ru1- Ru5** in ACN.

|  |  |
| --- | --- |
|  |  |
|  | |

**Figure S44.** Emissionspectra of complexes **Ru1-Ru5** in ACN.


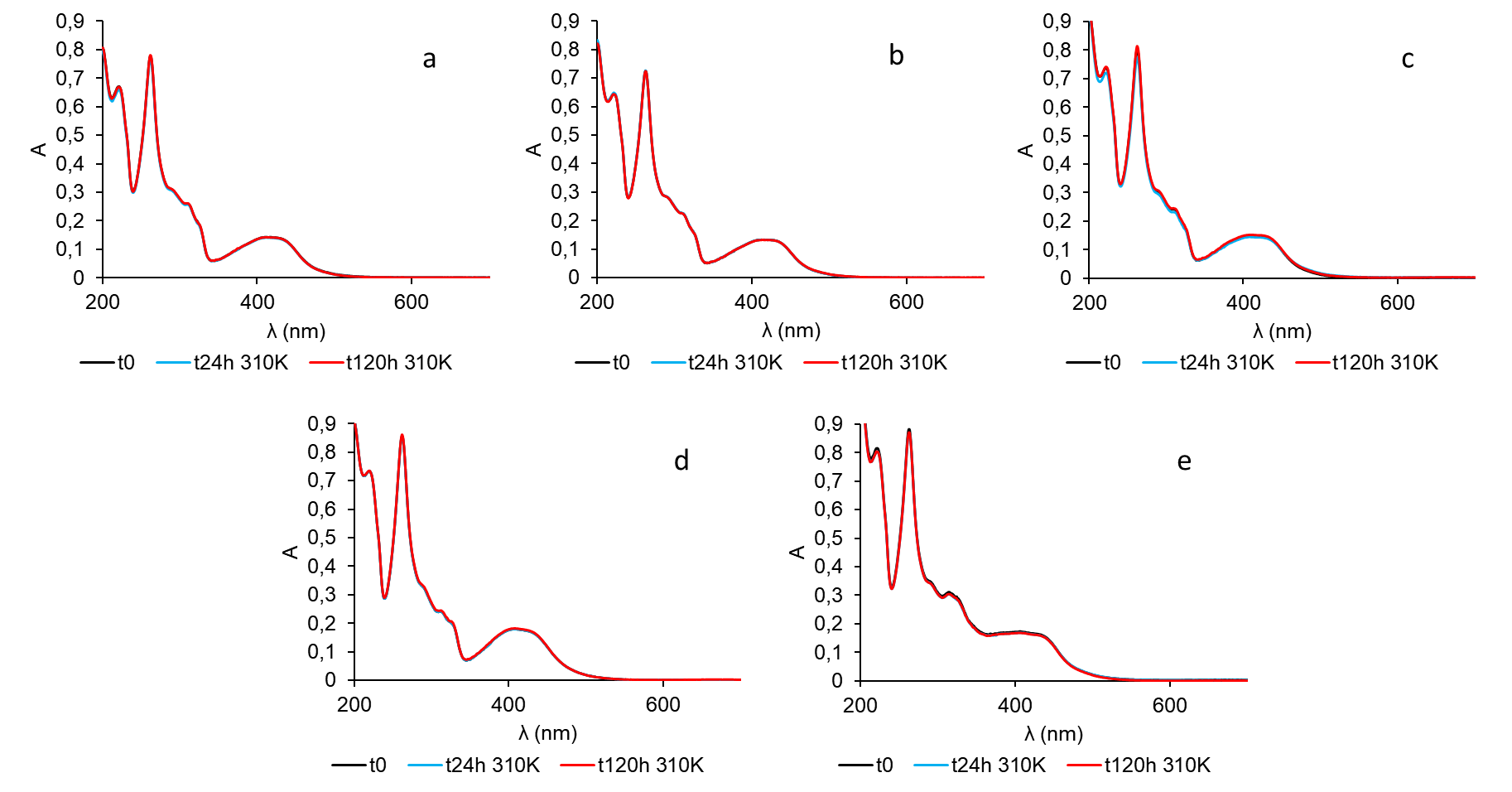


**Figure S45.** Stability of **Ru1** (a), **Ru2** (b), **Ru3** (c), **Ru4** (d) and **Ru5** (e) over 120 h in H_2_O at 310 K. t = 0 (grey), t = 24 h (blue) and t = 120 h (red line).


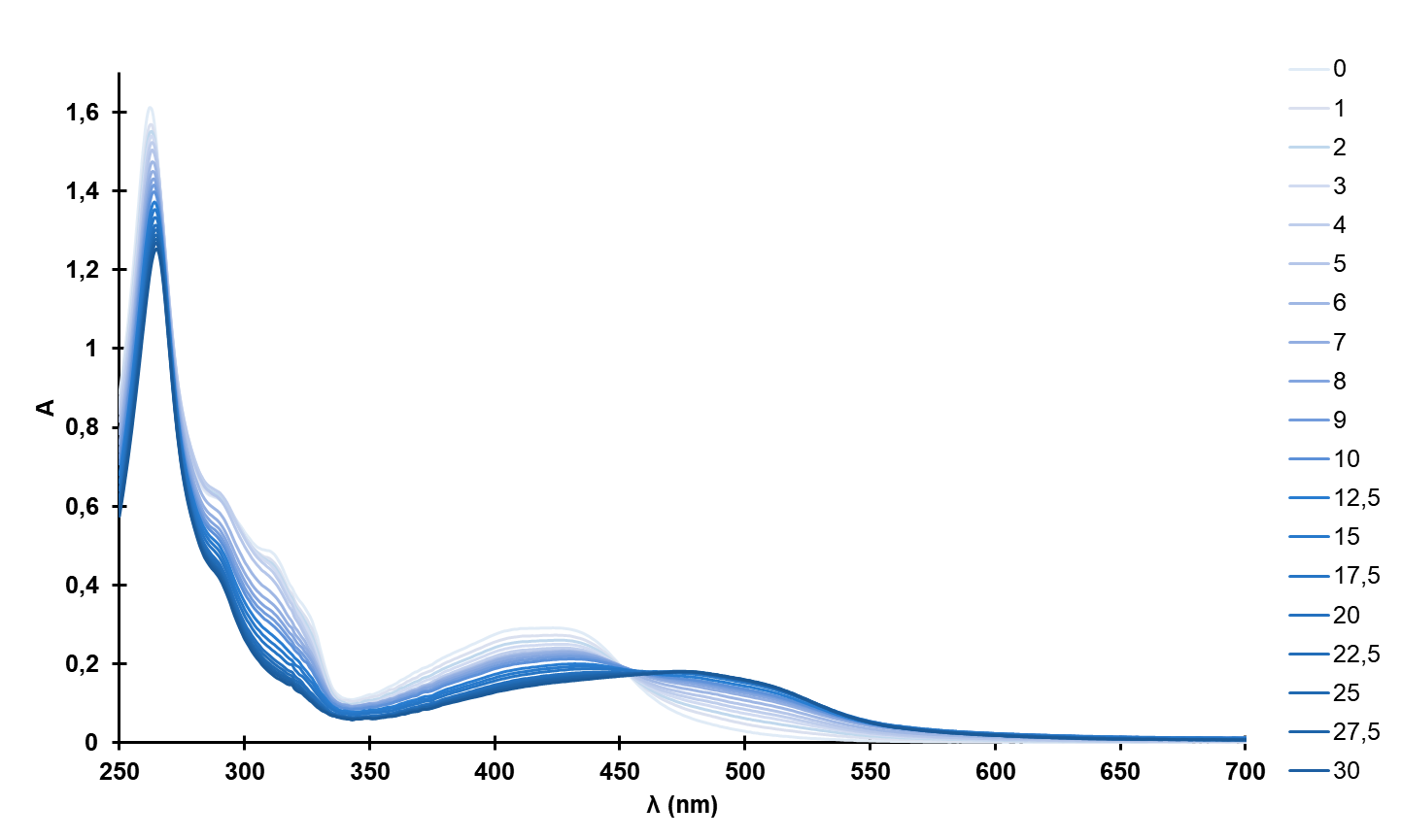


a


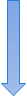

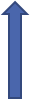

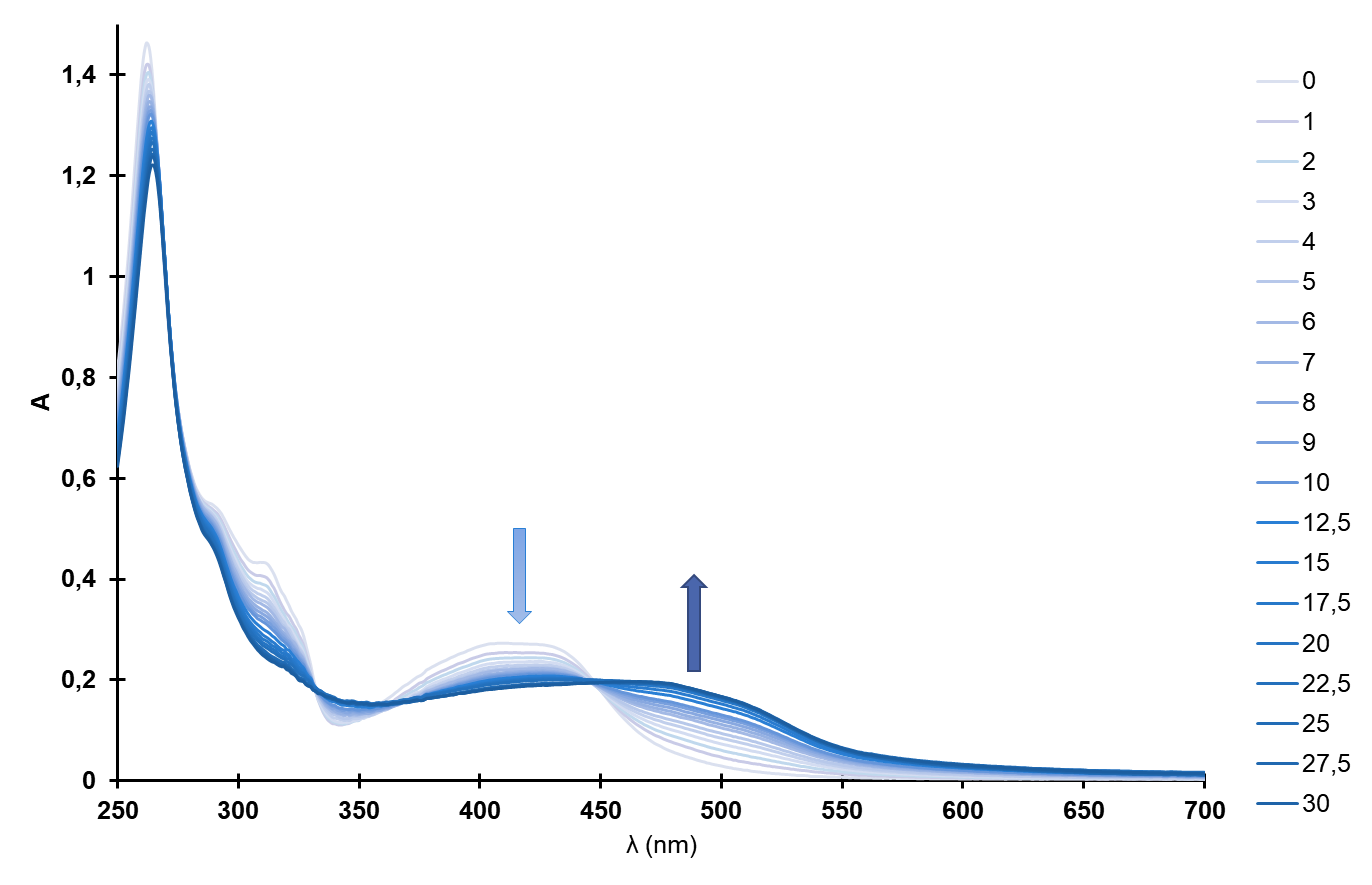


b


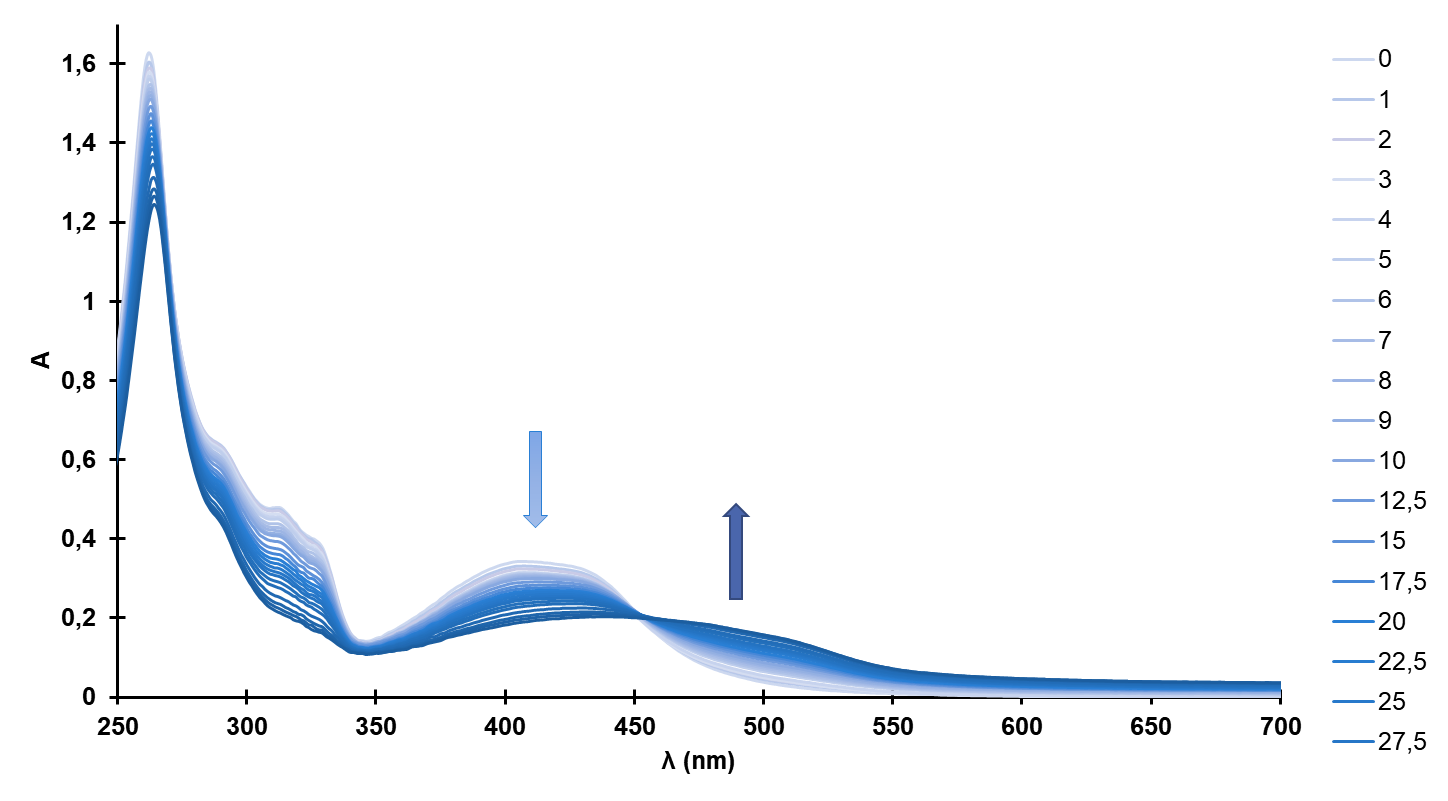


c


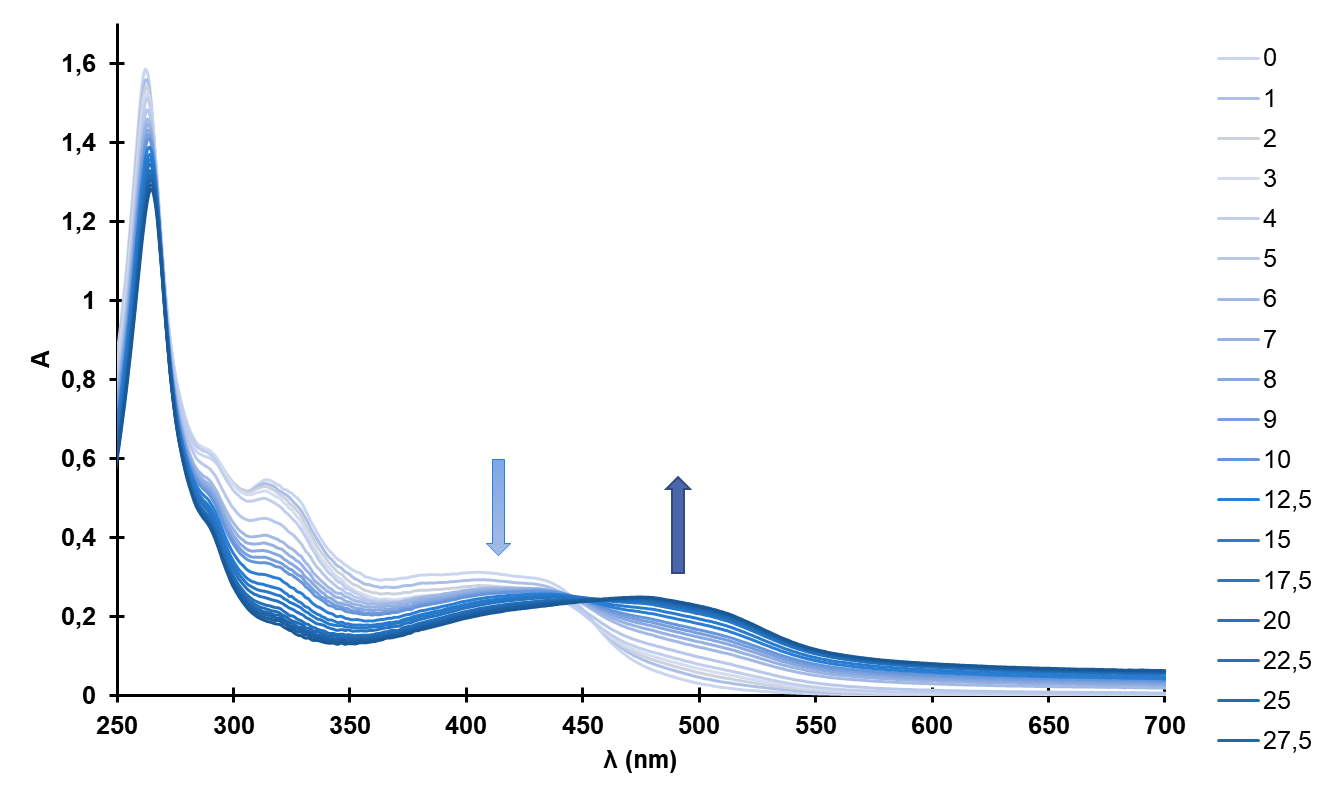


d


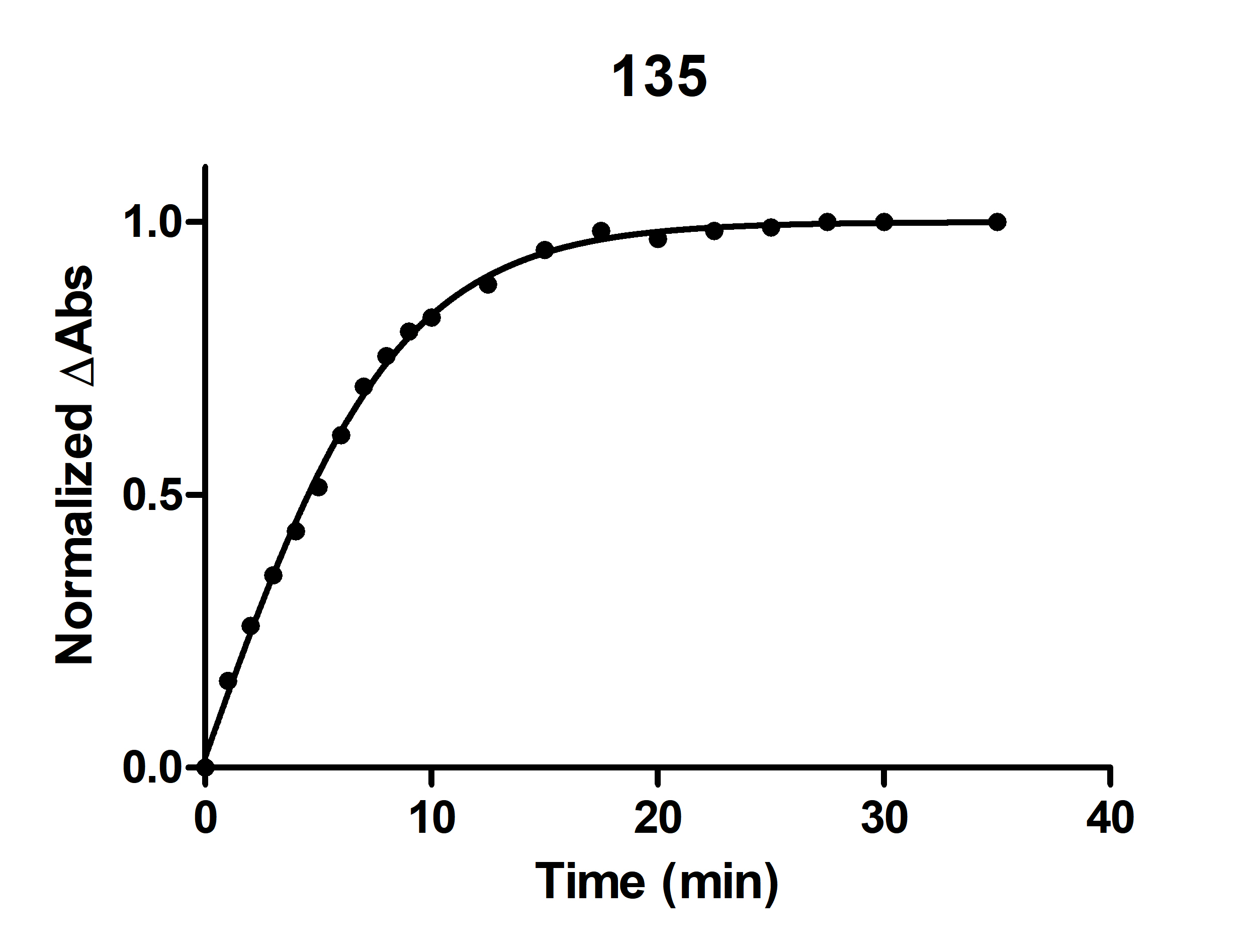

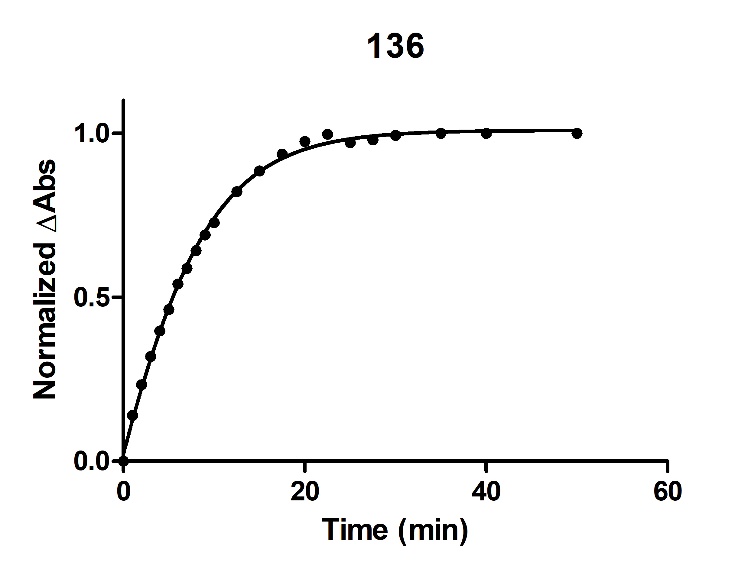

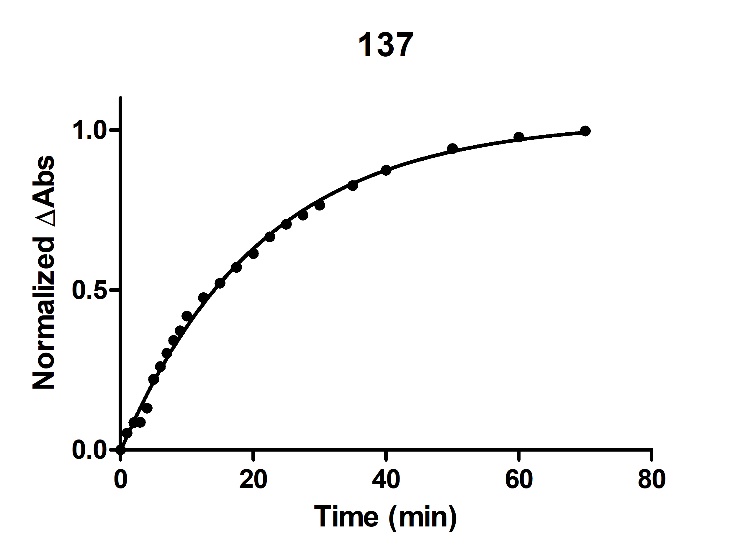

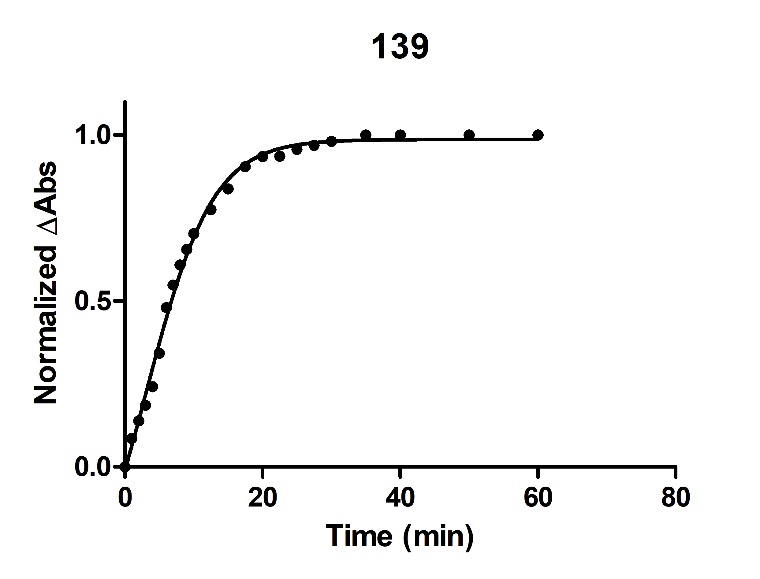


**Figure S46**. Changes in absorption spectra of **Ru2** (a), **Ru3** (b), **Ru4** (c) and **Ru5** (d) in H_2_O (10^-5^ M) as observed upon irradiation with blue ligth (λex = 465 nm, 3 mW/cm^2^). Arrows indicate the direction of the change in absorbance with increasing periods of irradiation in min (left) and photoejection kinetics (right).

**Figure S47**. Determination of photoejection products of **Ru1** by HPLC. HPLC chromatogram (DAD = 320 nm) of **Ru1** at t = 0 (A), after being irradiated for 1 h with blue light, λ_ex_ = 465 nm, 2 mW/cm^2^ (B) and the respective free ligand (C). The signal ~ 4.4 is an impurity of the solvent.


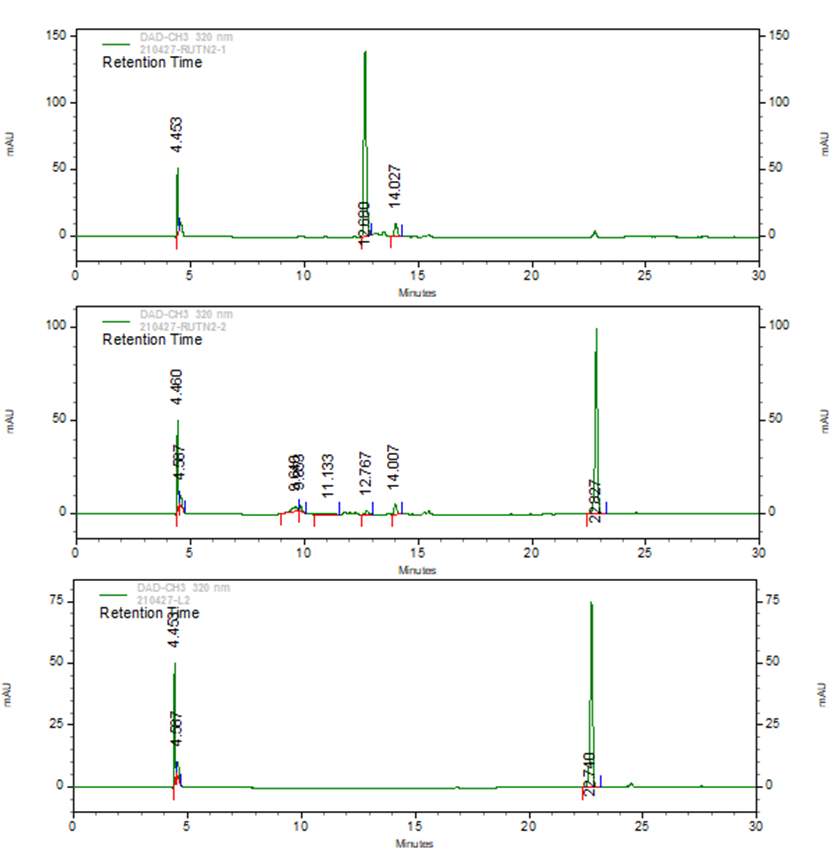


**A)**

**B)**

**C)**

**Figure S48**. Determination of photoejection products of **Ru2** by HPLC. HPLC chromatogram (DAD = 320 nm) of **Ru2** at t = 0 (A), after being irradiated for 1 h with blue light, λ_ex_ = 465 nm, 2 mW/cm^2^ (B) and the respective free ligand (C). The signal ~ 4.4 is an impurity of the solvent.

**Figure S49**. Determination of photoejection products of **Ru3** by HPLC. HPLC chromatogram (DAD = 320 nm) of **Ru3** at t = 0 (A), after being irradiated for 1 h with blue light, λ_ex_ = 465 nm, 2 mW/cm^2^ (B) and the respective free ligand (C). The signal ~ 4.4 is an impurity of the solvent.

**Figure S50**. Determination of photoejection products of **Ru4** by HPLC. HPLC chromatogram (DAD = 320 nm) of **Ru4** at t = 0 (A), after being irradiated for 1 h with blue light, λ_ex_ = 465 nm, 2 mW/cm^2^ (B) and the respective free ligand (C). The signal ~ 4.4 is an impurity of the solvent.

**Figure S51**. Determination of photoejection products of **Ru5** by HPLC. HPLC chromatogram (DAD = 320 nm) of **Ru5** at t = 0 (A), after being irradiated for 1 h with blue light, λ_ex_ = 465 nm, 2 mW/cm^2^ (B) and the respective free ligand (C). The signal ~ 4.4 is an impurity of the solvent.


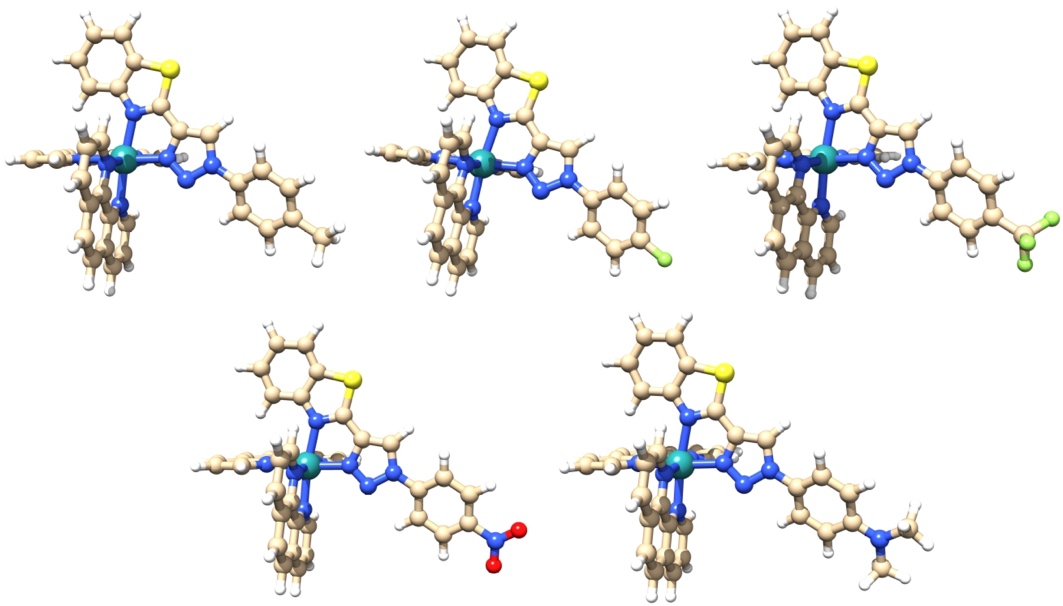


**Figure S52.** Structure of the Ru complexes **Ru1**- **Ru5** in water, singlet ground state, S_0_, obtained by DFT calculations.


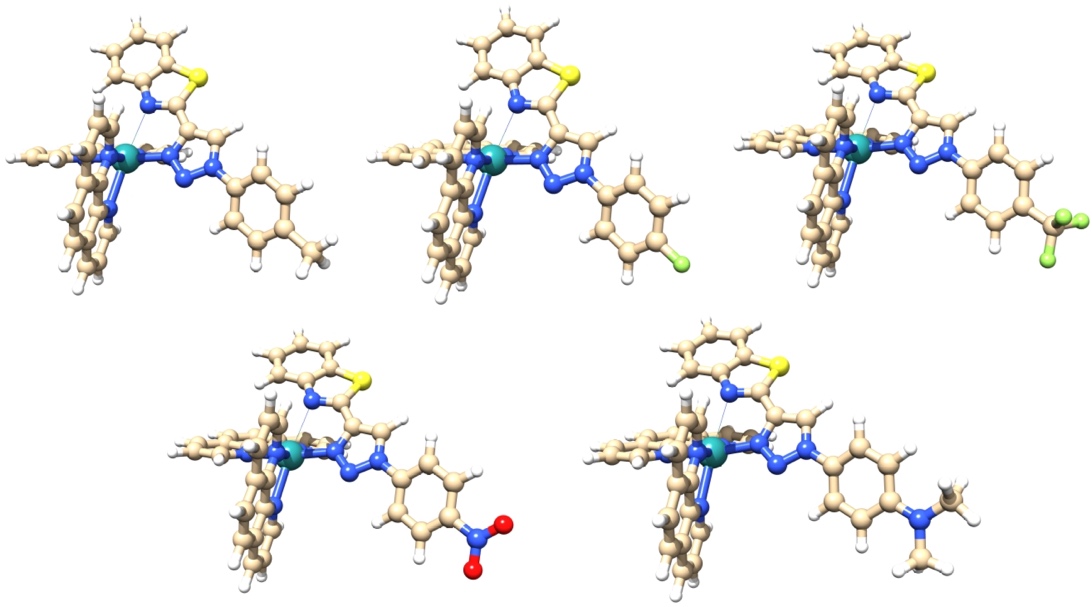


**Figure S53.** Structure of the Ru complexes **Ru1**- **Ru5** in water, triplet excited state, T_1_, obtained by DFT calculations.


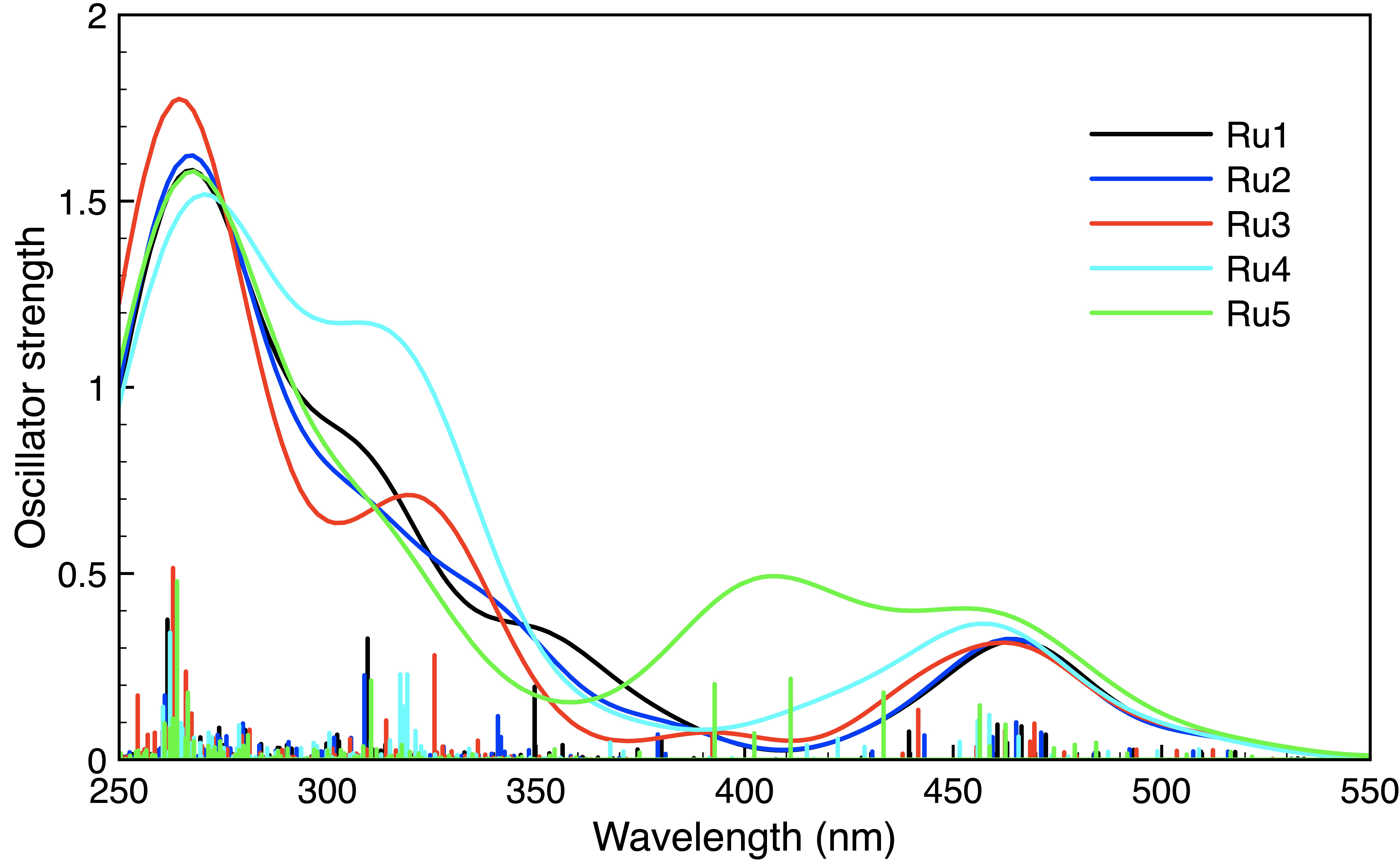


**Figure S54.** UV-visible absorption spectrum of the Ru complexes **Ru1** - **Ru5** in ACN, obtained by TD-DFT calculations.


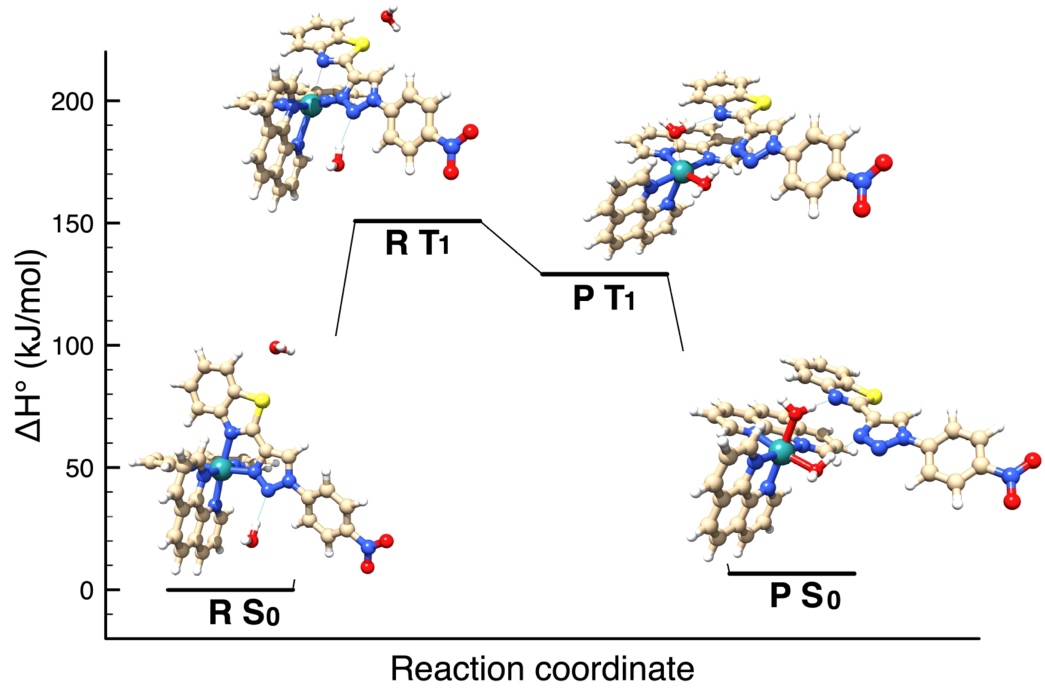


**Figure S55.** Proposed mechanism of ligand photo-ejection for the Ru complex **Ru4**, in H_2_O, obtained by DFT calculations: the ground state reactant, S_0_, in presence of two water molecules, is excited into the triplet state T_1_; in the excited state the two water molecules substitute the chelating ligand and finally the substitution product decays from T_1_ into the ground state S_0_.

**Table S1**. Crystal data and structure refinement for complexes **Ru1**, **Ru2** and **Ru5**.

| Complex | **Ru1** | **Ru2** | **Ru5** |
| --- | --- | --- | --- |
| Empirical formula | C_42_H_28_F_6_N_8_O_6_RuS_3_ | C_45_H_37_F_7_N_8_O_8_RuS_3_ | C_45_H_34_F_6_N_10_O_6_RuS_3_ |
| Formula weight | 1051.97 | 1148.07 | 1122.07 |
| Temperature / K | 100(2) | 100(2) | 100(2) |
| Wavelength / Å | 0.71073 | 0.71073 | 0.71073 |
| Crystal system | Triclinic | Triclinic | Triclinic |
| Space group | P-1 | P-1 | P-1 |
| Unit cell dimensions |  |  |  |
| *a* / Å | 13.1812(7) | 12.8869(12) | 13.0228(15) |
| *b /* Å | 13.2570(7) | 13.2041(13) | 14.4032(17) |
| *c /* Å | 15.2347(8) | 15.0619(14) | 15.1918(18) |
| *α  *° | 94.701(2) | 93.406(3) | 115.184(4) |
| *β  *° | 113.281(2) | 112.576(3) | 93.302(4) |
| *γ  *° | 94.484(2) | 96.730(3) | 92.744(4) |
| *V /* Å3 | 2419.3(2) | 2334.9(4) | 2566.1(5) |
| Z | 2 | 2 | 2 |
| Density_calcd_ / Mg/m3 | 1.444 | 1.633 | 1.452 |
| Absorption coefficient / mm-1 | 0.529 | 0.561 | 0.505 |
| F(000) | 1060 | 1164 | 1136 |
| Crystal size / mm3 | 0.170 x 0.070 x 0.030 | 0.230 x 0.050 x 0.020 | 0.090 x 0.080 x 0.040 |
| θrange for data collection / ° | 1.694 to 30.509 | 1.731 to 27.103 | 2.129 to 30.655 |
| Index ranges | -18 ≤ h ≤ 18  -18 ≤ k ≤ 18  -21 ≤ l ≤ 20 | -16 ≤ h ≤ 16  -16 ≤ k ≤ 16  -19 ≤ l ≤ 19 | -18 ≤ h ≤18 -20 ≤ k ≤ 20  -21 ≤ l ≤ 21 |
| Reflections collected | 37831 | 94868 | 191767 |
| Independent reflections | 14590 [R_int_ = 0.0556] | 10289 [R_int_ = 0.0467] | 15756 [R_int_ = 0.0594] |
| Completeness to θ = 30.000° | 99.0 % | 99.9 % ­ to θ= 25.242° | 100.0 % |
| Absorption correction | Semi-empirical from equivalents | Semi-empirical from equivalents | Semi-empirical from equivalents |
| Max. and min. transmission | 0.7461 and 0.6730 | 0.7457 and 0.6787 | 0.7410 and 0.7034 |
| Refinement method | Full-matrix least-squares on F2 | Full-matrix least-squares on F2 | Full-matrix least-squares on F2 |
| Data / restraints / parameters | 14590 / 163 / 669 | 10289 / 2 / 657 | 15756 / 110 / 711 |
| Goodness-of-fit on F2 | 1.068 | 1.107 | 1.070 |
| Final R indices  [I > 2σ(I)] | R_1_ = 0.0551  wR_2_ = 0.1149 | R_1_ = 0.0332  wR_2_ = 0.0782 | R_1_ = 0.0338  wR_2_ = 0.0869 |
| R indices (all data) | R_1_ = 0.0727  wR_2_ = 0.1219 | R_1_ = 0.0406  wR_2_ = 0.0854 | R_1_ = 0.0405  wR_2_ = 0.0914 |
| Largest diff. peak and hole / e.Å-3 | 1.230 and -0.990 | 1.880 and -0.693 | 0.898 and -0.743 |

**Table S2**. Hydrogen bonds for **Ru1** (Å and °).

| D-H...A | d(D-H) | d(H...A) | d(D...A) | <(DHA) |
| --- | --- | --- | --- | --- |
| C(18)-H(18)...O(68) | 0.95 | 2.38 | 3.127(4) | 135.7 |

**Table S3**. Hydrogen bonds for **Ru2** (Å and °).

_____________________________________________

| D-H...A | d(D-H) | d(H...A) | d(D...A) | <(DHA) |
| --- | --- | --- | --- | --- |
| O(63)-H(063)...O(93) | 0.909(19) | 1.984(19) | 2.892(3) | 177(4) |
| O(73)-H(073)...O(63) | 0.895(19) | 1.98(3) | 2.759(3) | 145(4) |
| C(5)-H(5)...O(83)#1 | 0.95 | 2.41 | 3.292(3) | 154.1 |
| C(9)-H(9)...O(73)#2 | 0.95 | 2.21 | 3.098(3) | 155.7 |

Symmetry transformations used to generate equivalent atoms:

#1 -x+1,-y,-z+2 #2 -x+1,-y+1,-z+1

**Table S4.** Selected π-π interaction parameters**.**

| **Complex** | **π-π**  **Interactions** | **Cg-Cg^a^**  **[Å]** | **α^b^**  **[º]** | **β^c^**  **[º]** | **Slippage^d^ [Å]** | **Symmetry operation on Cg** |
| --- | --- | --- | --- | --- | --- | --- |
| **Ru1**  Cg1 C32→C33→C34→C35→C39→C40  Cg2 C20→C21→C22→C23→C27→C28 | Cg1-Cg1 | 3.6063(19) | 0.00(15) | 19.7 | 1.217 | 1-X,2-Y,1-Z |
|  | Cg2-Cg2 | 3.6846(17) | 0.00(14) | 23.4 | 1.463 | 1-X,2-Y,-Z |
|  | Cg2-Cg3 | 3.7770(17) | 4.21(15) | 28.9 | 1.824 | 1-X,2-Y,-Z |
| **Ru2**  Cg1 C31→C32→C33→C34→C38→C39  Cg2 C19→C20→C21→C22→C26→C27  Cg3 N6→C22→C23→C24→C25→C26 | Cg1-Cg1 | 3.5938(14) | 0.03(11) | 22.1 | 1.354 | -X,1-Y,2-Z |
|  | Cg2-Cg2 | 3.6658(15) | 0.00(12) | 20.7 | 1.295 | X,1-Y,1-Z |
|  | Cg2-Cg3 | 3.7538(15) | 5.14(12) | 27.4 | 1.726 | -X,1-Y,1-Z |
| **Ru5**  Cg1 C33→C34→C35→C36→C40→C41  Cg2 C21→C22→C23→C24→C28→C29  Cg3 N8→C30→C31→C32→C33→C41 | Cg1-Cg1 | 3.5300(12) | 0.02(9) | 19.2 | 1.162 | -X,1-Y,1-Z |
|  | Cg2-Cg2 | 3.6218(11) | 0.02(8) | 19.8 | 1.228 | -X,1-Y,-Z |
|  | Cg1-Cg3 | 3.7029(11) | 4.16(9) | 28.6 | 1.770 | -X,1-Y,1-Z |

^a^ Distance between ring centroids.

^b^ Dihedral angle between planes I and J.

^c^ Angle between Cg(I)-Cg(J) vector and normal to plane I.

^d^ Distance between Cg(I) and perpendicular projection of Cg(J) on ring I.

**Table S5.** Bond distance (Å) and angles (˚) between the Ru atom and the coordinated nitrogen atoms of BTAT ligand in the Ru(II) octahedral complexes **Ru1** - **Ru5**, obtained from the X-ray structures and from the calculated structures obtained by DFT calculations in water (H_2_O) in the singlet ground state, S_0_, and in the first triplet excited state, T_1_.

|  | **X-ray structure** | | | **S_0_** | | | **T_1_** | | |
| --- | --- | --- | --- | --- | --- | --- | --- | --- | --- |
|  | Ru-N1 | Ru-N2 | N1-Ru-N2 | Ru-N1 | Ru-N2 | N1-Ru-N2 | Ru-N1 | Ru-N2 | N1-Ru-N2 |
| **Ru1** | 2.117 | 2.027 | 77.53 | 2.166 | 2.072 | 76.59 | 2.656 | 2.149 | 68.56 |
| **Ru2** | 2.117 | 2.036 | 77.91 | 2.166 | 2.072 | 76.6 | 2.654 | 2.149 | 68.54 |
| **Ru3** | - | - | - | 2.166 | 2.069 | 76.63 | 2.655 | 2.147 | 68.83 |
| **Ru4** | - | - | - | 2.165 | 2.066 | 76.69 | 2.709 | 2.144 | 68.24 |
| **Ru5** | 2.112 | 2.037 | 77.36 | 2.166 | 2.074 | 76.54 | 2.678 | 2.144 | 68.67 |

**Table S6.** Absorption wavelength (λ, nm), oscillator strength (f) and major contributions of occupied and virtual molecular orbitals (H: HOMO, L: LUMO), of the lower energy absorption transitions of compounds **Ru1** - **Ru5**, obtained by TD-DFT calculations in H_2_O solution (see Figure 4).

| Compound | Transition | λ (nm) | f | Major contributions (%) |
| --- | --- | --- | --- | --- |
| **Ru1** | S_0_→S_1_ | 586.50 | 0.0024 | H→L (70) |
| **Ru2** | S_0_→S_1_ | 584.38 | 0.0024 | H→L (70) |
| **Ru3** | S_0_→S_1_ | 579.21 | 0.0023 | H→L (70) |
| **Ru4** | S_0_→S_2_ | 724.89 | 0.0343 | H→L (70) |
| **Ru5** | S_0_→S_1_ | 681.38 | 0.0039 | H→L (70) |

**Table S7.** Dipole moments (Debye) of the Ru(II) octahedral complexes **Ru1**- **Ru5** in the ground state (μS_0_) and in the first triplet excited state (μT_1_), and their difference (μT_1_-μS_0_), obtained by DFT calculations, in water (H_2_O) and in acetonitrile (ACN).

|  | μS_0_ | | μT_1_ | | μT_1_-μS_0_ | |
| --- | --- | --- | --- | --- | --- | --- |
|  | H_2_O | ACN | H_2_O | ACN | H_2_O | ACN |
| **Ru1** | 2.41 | 2.43 | 3.54 | 3.56 | 1.13 | 1.13 |
| **Ru2** | 4.53 | 4.53 | 5.26 | 5.35 | 0.73 | 0.82 |
| **Ru3** | 12.02 | 12.01 | 12.69 | 12.70 | 0.67 | 0.69 |
| **Ru4** | 12.89 | 12.86 | 13.10 | 13.06 | 0.21 | 0.20 |
| **Ru5** | 2.42 | 2.43 | 3.63 | 3.53 | 1.21 | 1.09 |

**Table S8**. (Photo)toxicity test (normoxia/O_2_ 21%) of ligands **L1**- **L5**: [1 h treatment + 1 h irradiation under blue light].

|  | IC_50_ values (µM) | | | |
| --- | --- | --- | --- | --- |
|  | HeLa | | A375 | |
| Compound | Dark | Blue Light | Dark | Blue light |
| L1 | > 100 | > 100 | > 100 | > 100 |
| L2 | > 100 | > 100 | > 100 | > 100 |
| L3 | > 100 | > 100 | > 100 | > 100 |
| L4 | > 100 | > 100 | > 100 | > 100 |
| L5 | > 100 | > 100 | > 100 | > 100 |

**Table S9.** Resonances (ppm) of protons and carbons of the BTAT ligands

| H/C//complex | Ru1 | Ru2 | Ru3 | Ru4 | Ru5 |
| --- | --- | --- | --- | --- | --- |
| H2 | 8.4 | 8.4 | 8.4 | 8.4 | 8.4 |
| H3 | 7.45 | 7.44 | 7.47 | 7.47 | 7.45 |
| H4 | 7.10 | 7.10 | 7.11 | 7.11 | 7.09 |
| H5 | 5.90 | 5.91 | 5.91 | 5.91 | 5.91 |
| H9 | 10.61 | 10.68 | 10.89 | 10.59 | 10.18 |
| H11+H15 | 7.57 | 7.76 | 8.00 | 8.45 | 7.41 |
| H12+H14 | 7.37 | 7.44 | 7.94 | 7.94 | 6.78 |
| C1 | 150.7 | 150.7 | 150.7 | 150.7 | 150.6 |
| C2 | 125.1 | 125.0 | 125.1 | 125.1 | 124.9 |
| C3 | 127.0 | 127.0 | 127.1 | 127.2 | 126.9 |
| C4 | 128.2 | 128.2 | 128.2 | 128.3 | 128.1 |
| C5 | 118.2 | 118.2 | 118.3 | 118.3 | 118.2 |
| C6 | 134.2 | 134.2 | 134.3 | 134.4 | 134.1 |
| C7 | 159.4 | 159.3 | 159.2 | 159.1 | 159.4 |
| C8 | 144.4 | 144.4 | 144.7 | 145.0 | 144.0 |
| C9 | 126.7 | 126.7 | 127.1 | 126.7 | 125.4 |
| C10 | 133.4 | 132.2 | 130.5 | 139.7 | 124.7 |
| C13 | 140.1 | 162.3 | 130.0 | 148.1 | 151.0 |
| C11+C15 | 120.3 | 123.1 | 121.0 | 125.7 | 121.4 |
| C12+C14 | 130.3 | 116.9 | 127.4 | 121.4 | 111.9 |

**References**

(1) Ballester, F. J.; Ortega-Forte, E.; Bautista, D.; Santana, M. D.; Barone, G.; Ruiz, J. Newly Synthesized Benzothiazolyl-1,2,3-Triazole Derivatives: Intramolecular Charge Transfer Tuning, Solvatofluorochromism and Antiproliferative Properties. *Dyes and Pigments* **2023**, *209*, 110905. https://doi.org/10.1016/j.dyepig.2022.110905.
